# Self-esteem and peer pressure susceptibility mediating the link between maternal behavior and adolescent risk behaviors

**DOI:** 10.3389/fpsyg.2025.1655371

**Published:** 2025-11-06

**Authors:** Marija Milić, Vladimir Bjelobrk, Daniela Šincek

**Affiliations:** 1 Faculty of Humanities and Social Sciences, Josip Juraj Strossmayer University of Osijek, Osijek, Croatia; 2 Center for Autism, Osijek, Croatia

**Keywords:** parental psychological control, parental warmth, self-esteem, peer pressure susceptibility, internalizing and externalizing risk behaviors

## Abstract

**Introduction:**

Early adolescence is a crucial period of change, during which some youths begin to engage in externalizing and internalizing risk behaviors. Prevention at an early stage is vital to prevent more serious issues later. Although parental influence decreases and peer influence increases during this time, parental behavior still plays a significant role. This study examined the impact of parenting psychological control and warmth on the occurrence of risk behaviors in early adolescence, with susceptibility to peer pressure and self-esteem serving as potential mediators.

**Methods:**

The sample consisted of 410 eighth-grade students with an average age of 14 years. The instruments used were the Scale of Perception of Family Relationships, Susceptibility to Peer Pressure Scale, Rosenberg Self-Esteem Scale, and two subscales assessing risk-taking behavior among youth, which are part of the Self-Reported Risk and Delinquent Behavior Questionnaire.

**Results:**

The findings indicate that while models with both mediators included were confirmed, those in which peer pressure susceptibility mediated the relationship between parental behaviors and mild externalizing risk behaviors demonstrated a better fit. In contrast, in the relationship between parental behavior and internalizing risk behaviors, self-esteem emerged as a more effective mediator. All models showed partial mediation, indicating that only a portion of the influence of parenting behavior on the occurrence of risk behaviors is accounted for by these mediators. Analyses presented here focus on maternal data, with paternal results showing highly similar patterns.

**Discussion:**

The contribution of this study lies in the finding that parental warmth and psychological control and the development of mild externalizing and internalizing risk behaviors are mediated in somewhat different ways with respect to self-esteem and susceptibility to peer pressure. Aside from scientific implications, these results also have practical implications, as they suggest more concrete intervention strategies that can be implemented in family and school settings to mitigate risk behaviors.

## Introduction

Adolescence is a critical developmental stage characterized by profound and observable changes. During this period, individuals undergo substantial emotional, social, cognitive, and physical transformations as they strive to establish their identity. This process can be challenging for adolescents and their immediate environment, particularly their parents. Various factors influence it, determining whether it progresses smoothly or is accompanied by significant difficulties and potential deviations from normative behavior. This life stage is also characterized by an increased risk of engaging in risk behaviors. Whether adolescents engage in such behaviors depends on various factors, primarily family relationships, parental attitudes toward the child/adolescent, and the broader social environment in which they grow up.

Numerous theories explain the phenomenon of young people engaging in risk behaviors. One of the most well-known and frequently mentioned is Jessor's Theory of Problem Behavior (Jessor, 2016), which emerged in the mid-20th century. This theory offers a comprehensive socio-psychological conceptual framework for understanding problematic behavior among adolescents and young adults, grounded in the premise that behavior is shaped by the interaction between the individual and their environment.

Engagement in risk behaviors during adolescence can also be interpreted as an adaptive mechanism facilitating exploration in various relationships (especially peer relations and those with parents and siblings) and identity formation, representing the central developmental task of this period. For this reason, some researchers investigating youth risk behaviors have categorized specific actions—such as alcohol consumption, truancy, or fare evasion in public transportation—as undesirable normative behaviors (e.g., Ručević et al., 2009). The issue arises when these behaviors escalate into delinquent and violent conduct. To enable early intervention at the initial stages of delinquency development, it is crucial to identify the key factors contributing to the emergence and progression of risk behaviors that may later evolve into delinquent acts, particularly at the onset of adolescence.

Early adolescence is a unique developmental period marked by significant changes in relationships with parents and peers. While parents previously held a central role in adolescents' lives, their influence gradually diminishes as peer relationships become increasingly significant. However, parents remain a crucial source of support (Berk, 2015), and the nature of their relationship with their children—both before and during this period—plays a fundamental role in shaping adolescent behavior, peer selection, self-perception, and engagement in risky activities. The influence of family dynamics on adolescent involvement in risk behaviors has been well-documented in numerous prior studies (e.g., Hinnant et al., 2015; Kawabata et al., 2011; Novak et al., 2022; Raboteg-Šarić et al., 2002; Vieno et al., 2009).

The parent-child relationship has been identified as a key factor influencing the emergence of risk behaviors. Two fundamental dimensions define this relationship: emotional warmth and parental control (Baumrind, 1971). These dimensions form the basis for the classification of parenting styles, which describe characteristic patterns of parental behavior that shape children's development.

Classical parenting typologies (Baumrind, 1971; Maccoby and Martin, 1983), including authoritative, authoritarian, permissive/indulgent, and neglectful/uninvolved styles, represent combinations of two core dimensions: behavioral control and warmth. These typologies have been influential in shaping the field. However, they are also limited in several ways. By categorizing parenting into broad styles, these models risk oversimplifying the complex and reciprocal nature of parent–child interactions. Children are not passive recipients of parenting; instead, they actively shape parental behaviors, a dynamic that typological approaches often overlook. Furthermore, the cultural and societal context in which parenting occurs is not adequately addressed. For instance, Pinquart and Kauser (2018) emphasized that the benefits of the authoritative style, traditionally viewed as the most adaptive within Western middle-class families, do not generalize across all cultural settings. Studies with ethnic minority families in the United States have even suggested that authoritarian parenting may, in specific contexts, be linked with more favorable adjustment outcomes than the authoritative style (Chao, 1994, 2001; Deater-Deckard et al., 1996). However, more recent evidence suggests that authoritarian parenting is not necessarily adaptive in collectivistic or traditional societies. For example, Chen et al. (2024) reported that it was associated with poorer outcomes, including diminished self-esteem and a weaker self-concept. Similarly, a Spanish study by Palacios et al. (2022) has shown that permissive parenting was linked with outcomes comparable to, or even more positive than, those associated with authoritative parenting. In contrast, authoritarian and neglectful styles were consistently tied to less favorable health indicators. Recent research highlights the importance of parental psychological control in understanding adolescent adjustment. For example, studies have shown that higher levels of psychological control are linked to lower self-esteem and increased risky behaviors among adolescents (Bean et al., 2003; Nasiri Farsi and Pali, 2025). Previous research highlights that two parenting dimensions, psychological control and emotional warmth, are frequently associated with the emergence of adolescent risk behaviors. Consequently, when exploring the mechanisms underlying the development of such behaviors, it may be more informative to conceptualize parenting in terms of these dimensions rather than relying solely on broader parenting style classifications.

Emotional warmth encompasses closeness, acceptance, support, and attachment, whereas control involves supervision, restrictiveness, discipline, and demands for conformity (Maccoby and Martin, 1983). Parental control is generally conceptualized in two forms: behavioral and psychological. While behavioral control pertains to the regulation of a child's actions, psychological control has garnered increasing attention due to its potentially harmful effects. Defined by intrusive and manipulative parental behaviors—such as guilt induction, love withdrawal, and passive aggression—psychological control has been consistently linked to a range of adverse developmental outcomes, including both intrinsic risk behaviors—such as social withdrawal, persistent anxiety, and emotional distress—and extrinsic risk behaviors—such as aggression, alcohol, and substance abuse (Luk et al., 2017; Pettit et al., 2001; Smetana and Daddis, 2002). Parental psychological control in children aged 10 to 14 is positively linked to risk and aggressive behavior. In contrast, emotional warmth and supportive parenting are associated with a lower likelihood of engagement in risky activities, higher self-esteem, and fewer emotional difficulties, such as depression (Finkenauer et al., 2005).

Although most research on parenting and adolescent risk behaviors has been conducted in Anglo-American and Western European societies, it is essential to consider how these dynamics unfold in other cultural contexts. Parenting is shaped not only by individual beliefs but also by broader social norms and expectations that guide family life and influence children's development. In Croatia, for example, the traditional importance of strong family bonds and close relationships between parents and children gives parenting a particular significance, often different from what is seen in more individualistic contexts. Researching these topics in Croatia reveals how local culture influences parenting and adolescent behavior, providing new perspectives that extend beyond Western approaches. For example, research (Brust Nemet and Vrdoljak, 2022; Kero, 2022) indicates that in Croatia, mothers are predominantly responsible for caring for children and are mainly involved in leisure and recreational activities (Berc and BlaŽeka Kokorić, 2012). (Berc and BlaŽeka Kokorić 2012) concluded that such patterns reflect prevailing gender stereotypes within the sociocultural context, where traditional expectations still shape parental roles, and fathers are less frequently expected to engage actively with their children, particularly the younger ones. In Croatia, traditional values emphasizing family cohesion and interdependence remain strong, as reflected in the tendency of young people to live with their parents for more extended periods compared to Western countries. At the same time, individualistic values—shaped by Western cultural influences and social change—are becoming increasingly prominent, highlighting autonomy and self-realization. The coexistence of these collectivistic and individualistic orientations influences how parenting roles and family relationships are experienced (Gvozdanovic et al., 2019).

In addition to parental behavior, self-perception may also be associated with involvement in risk behaviors. Self-esteem is an individual's awareness of their worth (Harter, 1999). Although it represents a personal evaluation, it is influenced by interactions with others. Individuals evaluate themselves by comparing their own traits and behaviors to those of others, including how they believe others perceive them. Reflected appraisals, social comparisons, and self-attributions (Rosenberg et al., 1989) represent central factors in the development of self-esteem. Given these influences, it logically follows that the parent-child relationship plays a significant role in shaping children's self-esteem. Since individuals strive to maintain a high level of self-esteem, they engage in behaviors they believe will enhance their self-worth. A substantial body of research has established a negative association between self-esteem and risk behaviors, a relationship that is also evident among early adolescents (Martínez-Casanova et al., 2024; Rosenberg et al., 1989). Upon entering adolescence, young individuals become particularly vulnerable to declines in self-esteem. This period is marked by the shift from primary to secondary education, cognitive transformations, and emotional turbulence, all contributing to decreased self-esteem (Hirsch and DuBois, 1991).

Given the increasing influence of peers during this stage, some adolescents may engage in risk behaviors to restore or enhance their self-worth, particularly in the context of relationships with peers and friends. This tendency is particularly evident among individuals who are more susceptible to peer pressure (Tian et al., 2020). Peers are adolescents' preferred source of support (Dopp and Cain, 2012). Susceptibility to peer pressure arises from the adolescent's need for group belonging (Brown et al., 1986), particularly during this developmental stage (Brendt et al., 1989, as cited in Lebedina-Manzoni et al., 2008). Peer pressure refers to the expectation that individuals conform to specific behaviors, regardless of personal preferences, to gain social acceptance within a desired group (Lebedina-Manzoni et al., 2008). Susceptibility to peer pressure has been linked to numerous externalizing risk behaviors such as the consumption of alcoholic beverages (Forko and Lotar, 2012), cigarettes, and psychoactive substances, as well as early engagement in sexual activity (KneŽević Florić et al., 2021; Lebedina-Manzoni et al., 2008) and violent behaviors (Ãuranović and Klasnić, 2016). Ajduković et al. (2008) report that adolescents engaging in risk behaviors are likelier to choose peers who exhibit similar behaviors, as peer pressure toward socially unacceptable conduct significantly predicts the adoption of maladaptive normative behaviors. Although most research has focused on examining the influence of peer pressure on the emergence of externalizing behaviors, susceptibility to peer pressure has also been confirmed in the development of internalizing risk behaviors (Heilbron and Prinstein, 2008; Prinstein et al., 2010). Susceptibility to peer pressure plays a role in self-injury, with evidence suggesting that adolescents are more likely to engage in non-suicidal self-injury if their peers do (Prinstein et al., 2010; James, 2013). Several theoretical frameworks may account for this association. According to social learning theory, adolescents adopt behaviors by observing their peers (Heilbron and Prinstein, 2008), while identity theories emphasize the need for acceptance, thereby increasing susceptibility to peer influence (Heilbron and Prinstein, 2008). Attraction theories propose that adolescents tend to choose friends who are similar to themselves, thereby reinforcing behaviors such as self-injury within peer groups (Byrne, 1971, 1997; as cited in Heilbron and Prinstein, 2008).

Weaker ties with prosocial peers may increase the likelihood of forming stronger connections with those engaged in risk behaviors (Novak et al., 2022). Rejected adolescents, driven by the need for social acceptance, are especially vulnerable to affiliating with deviant peer groups (Osgood et al., 2013).

As previously noted, behavioral problems are typically categorized as internalizing and externalizing but are often interrelated (Maglica and DŽanko, 2016). The concepts of internalizing and externalizing problems were first introduced in 1966, following a factor-analytic study of psychiatric symptoms in children (Achenbach, 1966). Both are highly prevalent in youth (Achenbach et al., 2003) and linked to adverse outcomes, including poor academic performance, peer difficulties, delinquency, and compromised mental health (Champion et al., 1995; Fergusson et al., 2005).

Internalizing problems are often manifestations of affective states, such as anxiety, depression, withdrawal, and social difficulties. They are directed inward and can be challenging to detect (Bouillet and Bijedić, 2007; Lebedina-Manzoni, 2007). Children with internalizing behavioral problems may exhibit tension, shyness, fears, sadness, withdrawal, anxiety, and depression (Lebedina-Manzoni, 2007), difficulties in social adaptation, and even suicidal tendencies (Maglica and DŽanko, 2016). Internalizing problems are often associated with school dropout, substance use, and suicidality, and they carry the most significant adverse consequences for an individual's later life (Liu et al., 2011). To explain why some adolescents are particularly vulnerable to these outcomes, researchers have examined a range of contributing factors, including family dynamics, adverse life events, genetic predispositions, and broader social influences. Research highlights familial influences in the development of depression (Tully et al., 2008) and anxiety (Biederman et al., 2001), with adverse life events such as violence, poverty, abuse, parental separation, and bereavement further increasing vulnerability (Reinherz et al., 1999; Toth and Cicchetti, 1996). Moreover, dysfunctional family communication and conflict have also been identified as key risk factors for suicidal behavior in adolescence (Bilsen, 2018). Low-income family communication is often linked to suicide, both in relation to the child's problems and in general family interactions. Conflicts with parents, as well as a lack of communication, are identified as key factors (Gould et al., 1996; Portzky et al., 2005).

Externalizing problems encompass a range of disruptive behaviors, including hyperactivity, oppositionality, aggressive behavior, antisocial tendencies, rule-breaking, school truancy, theft, robbery, substance abuse, and delinquency, often described in the literature as conduct problems or antisocial behavior (Hinshaw, 1987; Zelenika et al., 2024; ŽiŽak et al., 2004). These externalizing risk behaviors can be understood as a group of difficulties that manifest primarily in the child's interactions with the external environment (Campbell et al., 2000). While childhood externalizing issues may initially appear less severe, they often serve as precursors to more serious conditions, such as conduct disorder and delinquency in adolescence and adulthood (Farrington, 1997; Liu, 2004). Developmental models emphasize the interaction of biological vulnerabilities (e.g., genetic predispositions and prenatal complications) with psychosocial risks, such as family stress, hostile parenting, and social adversity, in shaping pathways toward later aggression, hyperactivity, and delinquent behavior (Jessor, 2016; Loeber and Farrington, 2000; Raine et al., 1997).

Based on previous research, the present study hypothesizes that self-esteem and susceptibility to peer pressure may mediate the influence of parental emotional warmth and psychological control on involvement in externalizing and internalizing risk behaviors.

Previous research has investigated the influence of parental control and warmth on self-esteem (Krauss et al., 2020; Zhang et al., 2024a,b), as well as susceptibility to peer pressure (Chan and Chan, 2013). Moreover, parental warmth and psychological control have been identified as significant predictors of adolescents' engagement in risk behaviors (Jackman and MacPhee, 2017; Novak et al., 2022; Vieno et al., 2009). Additionally, studies have examined the relationship between self-esteem and susceptibility to peer pressure (Omisola et al., 2022). However, there is a lack of research focusing on younger adolescents that explores a model in which these two factors mediate the relationship between parental control and warmth, as well as the occurrence of risk behaviors in this age group. Previous research has demonstrated the mediating role of self-esteem between authoritative parenting style and aggression (Hesari and Hejazi, 2011) among undergraduate female students, as well as the influence of parenting style and susceptibility to peer pressure on risk behaviors among youth aged 15 to 25 (Aluko et al., 2024). Additionally, the fact that the parent-child relationship is associated with the occurrence of risk behaviors in children has been well established by numerous studies (i.e., Hinnant et al., 2015; Kawabata et al., 2011; Novak et al., 2022; Pettit et al., 2001; Raboteg-Šarić et al., 2002; Smetana and Daddis, 2002; Vieno et al., 2009). However, this research aims to examine the mechanisms through which this relationship operates. Specifically, it aims to determine whether these mechanisms differ in the emergence of internalizing and externalizing risk behaviors among early adolescents.

## Materials and methods

### Participants

The study was carried out among eighth-grade students across all 15 elementary schools in the city of Osijek, Croatia. In the Croatian education system, primary education spans eight years and typically begins at the age of six or seven. For greater clarity in an international context, the final 4 years of primary education can be characterized as “lower secondary school” or “middle school.” In total, 410 students participated in the study (218 female and 181 male students, with 11 participants not specifying their gender). The age range of the participants was from 13 to 16 years, with a mean age of 14 years (*M* = 14.2; *SD* = 0.44). Overall, 62% of all eighth-grade students in the city of Osijek participated in the study. The study adhered to the guidelines of the Ethics Code for research with children.

### Variables and measures

#### Parental emotional warmth and psychological control

The Scale of Perception of Family Relationships (Macuka, 2004), which measures bipolar dimensions of emotional warmth and psychological control, was used. The scale comprises 25 items, with 15 items assessing parental warmth toward the child and 10 evaluating parental psychological control. Participants rated each item on a three-point scale (from 1 to 3) regarding how accurate each item was for them, with values ranging from “*not at all true*” to “*completely true*”), for both their mother and father (separately). The total score for each subscale was calculated by summing the points for each item. A higher score on the Parental Emotional Warmth subscale indicates greater closeness and acceptance, while a higher score on the Psychological Control subscale indicates more control and criticism of the child's behavior. The internal reliability (Cronbach's alpha) for the individual subscales is as follows: maternal emotionality, Cronbach's alpha = 0.88; paternal emotionality, Cronbach's alpha = 0.92; maternal control, Cronbach's alpha = 0.83; paternal control, Cronbach's alpha = 0.85.

### Peer pressure susceptibility

Susceptibility to peer pressure was examined using an adapted version of the Susceptibility to Peer Pressure Scale by Lebedina-Manzoni et al. (2008). The scale was adapted by modifying the items to reflect participants' experiences instead of hypothetical situations. The scale consists of 22 items, which participants rated on a scale from 1 to 5 regarding how accurate each item was for them, with values ranging from “*not at all true*” to “*completely true*.” A higher score indicates greater susceptibility to peer pressure. The reliability of the scale, expressed by Cronbach's alpha, was 0.84.

### Self-esteem

Self-esteem was measured using the Rosenberg Self-Esteem Scale (Rosenberg, 1965), which includes ten items (five positive and five negatively worded). It has a Likert-type answering scale with four response options, ranging from “*strongly disagree*” to “*strongly agree*.” The total score is the sum of all items, with reverse scoring for the negatively worded items. Scores range from 10 to 40, with higher scores indicating higher self-esteem. In this study, Cronbach's alpha was 0.89.

### Risk behaviors

Risk-taking and delinquent behavior were measured using the Self-Reported Risk and Delinquent Behavior Questionnaire (SRDP) (Ručević et al., 2009). The questionnaire assesses behavior frequency (0- never, 1- 1–2 times, 2- 3–5 times, 3- more than 5 times) through 43 items across seven factors: (1) misdemeanor and minor delinquent behaviors, (2) undesirable normative behaviors; (3) risky sexual behaviors; (4) drug abuse; (5) violence in close relationships; (6) serious delinquency—theft, burglary, and robbery; and (7) suicidal and self-aggressive behaviors. Factors 2, 3, and 7 represent risk behaviors, while the others measure delinquency. Each item has a severity index (1–9) (Ručević et al., 2009). For example, “*Skipping school*” is rated 1, while “*Hitting or seriously injuring a teacher*” is rated 9. Subscale scores were calculated as the sum of weighted item scores. The personal result for each item is calculated by multiplying the item's severity index (1–9) by the personal frequency (0–3). Six subscales had acceptable reliability (0.60–0.78), while Violence in close relationships was 0.39 and was excluded from further analyses. In this study, two subscales from the questionnaire were used: one to assess mild extrinsic risk behaviors (Undesirable normative behaviors) and another to examine internalizing risk behaviors (Suicidal and self-aggressive behaviors), with both subscales focusing on risk behaviors that do not constitute legal offenses.

### Procedure

Ethical approval for the study was obtained from the Ethics Committee of the Faculty of Humanities and Social Sciences in Osijek. School principals in the city of Osijek were contacted with detailed information about the study and a request for their students' participation. Upon receiving approval from the principals, parental consent forms were distributed. Students whose parents provided written consent participated in the study during the regular class teacher period. The research was administered in groups, lasting one class period, and was conducted by the researcher in all classes. Participants were informed about the study's purpose, their voluntary and anonymous participation, and their right to withdraw at any time. They were also assured that the collected data would be used exclusively for research purposes and analyzed at the group level. To ensure privacy, students sat individually, with physical partitions used when necessary. Instructions were read aloud before completing the questionnaires. The order of questionnaire administration was rotated across classes according to a Latin square design to control for order effects. After completion, participants were thanked and advised to seek support from school counseling services if they experienced any distress during the process.

### Data analysis

Data were analyzed using IBM SPSS Statistics 20.0 and the PROCESS macro version 3.3 (Hayes, 2013). Preliminary analyses included descriptive statistics (means and standard deviations) and correlation coefficients to examine associations between the study variables. Before conducting inferential analyses, the assumptions of normality, linearity, homoscedasticity, and absence of multicollinearity were verified and found to be satisfied. The measures of intrinsic and extrinsic risk behaviors, assessed with the SRDP subscales Undesirable Normative Behaviors and Suicidal and Self-Aggressive Behaviors, showed deviations from normality criteria. Following previous research conducted by the scale's developers and other studies using these subscales (Livazović and Ručević, 2012; Ručević, 2011), as well as statistical recommendations (Field, 2013; Howell, 2007; Tabachnick and Fidell, 2013), logarithmic transformations were applied to these subscale scores. After the transformations, the skewness and kurtosis indices were within acceptable ranges.

To test the hypothesized mediational processes, we used Hayes' PROCESS macro, Model 6, which specifies serial multiple mediation. In this model, the independent variable (X) predicts the dependent variable (Y) through two mediators (M1 and M2) operating in sequence. Gender was included as a control variable to account for potential demographic effects. The analyses were based on 5,000 bias-corrected bootstrap resamples, providing 95% confidence intervals for indirect effects. Effect sizes for mediation were reported using completely standardized indirect effects. All tests were two-tailed, and significance was set at *p* < 0.05.

## Results

### Correlation analysis of major study variables

Table 1 shows the means, standard deviations, and correlation coefficients for maternal and paternal psychological control, emotional warmth, self-esteem, peer pressure susceptibility, mild externalizing risk behaviors, and internalizing risk behaviors. Maternal psychological control exhibited a positive correlation with both externalizing (*r* = 0.33, *p* < 0.01) and internalizing risk behaviors (*r* = 0.38, *p* < 0.01), as did paternal psychological control (*r* = 0.32, *p* < 0.01 for mild externalizing risk behaviors, and *r* = 0.41, *p* < 0.01 for internalizing risk behaviors). Maternal warmth showed a negative correlation with both externalizing (*r* = 0.33, *p* < 0.01) and internalizing risk behaviors (*r* = 0.38, *p* < 0.01), as did paternal warmth (*r* = 0.28, *p* < 0.01 for mild externalizing risk behaviors and *r* = 0.48, *p* < 0.01 for internalizing risk behaviors). Self-esteem correlates negatively with parental psychological control (both maternal and paternal) and risk behaviors and positively with parental emotional warmth. On the contrary, peer pressure susceptibility is negatively correlated with self-esteem and parental emotional warmth and positively correlated with parental psychological control and risk behaviors. Although the SRDP scale is designed to assess a broad range of risk and delinquent behaviors, in the context of the present study, the focus was narrowed to include only those behaviors that are considered risky yet do not meet the threshold for legal punishment. Specifically, the analysis focused on mildly risky externalizing behaviors, such as minor rule-breaking or defiance, as well as internalizing risk behaviors, including self-harming.

**Table 1:** Means, standard deviations, and Pearson's correlation coefficients of all variables.

**Variables**	**M ±SD**	**1**.	**2**.	**3**.	**4**.	**5**.	**6**.	**7**.	**8**.	**9**.
1. Gender	1.55 ± 0.50	1								
2. Maternal psychological control	14.55 ± 3.96	0.14^**^	1							
3. Paternal psychological control	14.18 ± 4.11	0.15^**^	0.60^**^	1						
4. Maternal emotional warmth	40.68 ± 5.14	−0.17^**^	−0.65^**^	−0.47^**^	1					
5. Paternal emotional warmth	38.31 ± 6.86	−0.21^**^	−0.39^**^	−0.64^**^	0.61^**^	1				
6. Self-esteem	2.95 ± 0.67	−0.30^**^	−0.31^**^	−0.32^**^	0.39^**^	0.48^**^	1			
7. Peer pressure susceptibility	1.41 ± 0.43	0.13^*^	0.38^**^	0.35^**^	−0.38^**^	−0.33^**^	−0.28^**^	1		
8. Mild externalizing risk behaviors	6.03 ± 6.84	0.02	0.33^**^	0.32^**^	−0.29^**^	−0.28^**^	−0.17^**^	0.44^**^	1	
9. Internalizing risk behaviors	4.64 ± 10.72	0.26^**^	0.38^**^	0.41^**^	−0.47^**^	−48^**^	−0.45^**^	0.38^**^	0.29^**^	1

^*^p < 0.05, ^**^p < 0.01; Gender: male = 1, female = 2.

To investigate the hypothesized mediation mechanisms, a total of eight regression-based mediation analyses were conducted using Hayes' PROCESS macro. Specifically, we examined whether maternal and paternal psychological control and emotional warmth predicted adolescents' externalizing and internalizing risk behaviors through two theoretically relevant mediators: self-esteem and peer pressure susceptibility. The analyses consistently demonstrated that the pattern of associations, including both significant and non-significant paths, was identical across mothers and fathers. Moreover, the explained variance (*R*^2^) in the regression analyses was highly similar, with differences typically within 3–4 percentage points and at most up to 6 percentage points in one analysis. Given this substantial overlap, and to avoid redundancy, only the maternal models are reported in the main text. Maternal results were selected as the reference point because prior research (e.g., Lebedina-Manzoni and Ricijaš, 2013; Wu et al., 2022; Zhang et al., 2024a,b) has more frequently emphasized maternal influences on adolescent adjustment. The corresponding paternal models and results are provided in Supplementary material.

### Test of the mediating effects of self-esteem and peer pressure susceptibility between maternal psychological control and mild externalizing risk behaviors

Preliminary independent samples *t*-tests were conducted to examine potential gender differences in the study variables. The results showed that girls scored significantly higher than boys on internalizing risk behaviors, *t*_(365)_ = −5.34, *p* < 0.05, while no significant gender differences were found for mild externalizing risk behaviors. Given the observed gender differences in one of the outcome variables, as well as prior research indicating that gender may influence both internalizing and externalizing problems, gender was included as a control variable in all subsequent analyses to ensure a more accurate estimation of the hypothesized effects. After controlling for participants' gender, the results showed that maternal psychological control (β = 0.33, *p* < 0.001) had a positive impact on mild externalizing risk behaviors. After accounting for mediators, the direct effect was reduced (β = 0.19, *p* < 0.001), indicating that although part of the relation can be explained by self-esteem and peer pressure susceptibility as mediators, some part of the relation remains unexplained. Maternal psychological control significantly and negatively predicted self-esteem (β = −0.29, *p* < 0.001), indicating that higher levels of maternal psychological control are associated with lower self-esteem.

Additionally, it positively predicted peer pressure susceptibility (β = 0.32, *p* < 0.001), indicating that adolescents whose parents exhibit higher levels of psychological control are more susceptible to peer influence. On the other hand, self-esteem did not significantly predict undesirable normative behavior; however, it did significantly negatively predict peer pressure susceptibility (β = −0.18, *p* < 0.001), suggesting that lower self-esteem is associated with a higher susceptibility to peer influence. Peer pressure susceptibility significantly predicts risk behaviors (β = 0.35, *p* < 0.001), highlighting its key role in explaining adolescents' engagement in risk behaviors (Table 2).

**Table 2:** Regression model of the effect of maternal psychological control on adolescents' mild externalizing risk behaviors.

**Variables**	**β**	***t***	***p***	***R* ^2^**	***F***
**Step 1 outcome variable: Self-esteem**					
Control variable: Gender	−0.26	−5.53	0.00	0.17	40.583^*^
Predictor: Psychological control	−0.29	−6.29	0.00		
**Step 2 outcome variable: Peer pressure susceptibility**					
Control variable: Gender	0.03	0.70	0.48	0.18	27.961^*^
Predictor: Psychological control	0.32	6.51	0.00		
Mediator 1: Self-esteem	−0.18	−3.54	0.00		
**Step 3 outcome variable: mild externalizing risk behaviors**					
Control variable: Gender	−0.05	−1.13	0.26	0.22	27.498^*^
Predictor: Psychological control	0.20	3.90	0.00		
Mediator 1: Self-esteem	−0.02	−0.34	0.73		
Mediator 2: Peer pressure susceptibility	0.36	7.27	0.00		

^*^p < 0.001.

In the first model (Table 2), with self-esteem as the outcome, the predictors explained 17% of the variance (*R*^2^ = 0.17, *F* = 40.58, *p* < 0.001), which represents a medium effect size (Cohen, 1988). Both gender and maternal psychological control were significant, with psychological control showing a more substantial negative effect on self-esteem.

In the second model, with peer pressure susceptibility as the outcome, the predictors accounted for 18% of the variance (*R*^2^ = 0.18, *F* = 27.96, *p* < 0.001). This again reflects a medium effect (Cohen, 1988). Here, maternal psychological control was positively associated with susceptibility to peer pressure, while self-esteem made a negative contribution, indicating that self-esteem partially mediates the relationship between psychological control and peer pressure susceptibility.

**Figure 1:**
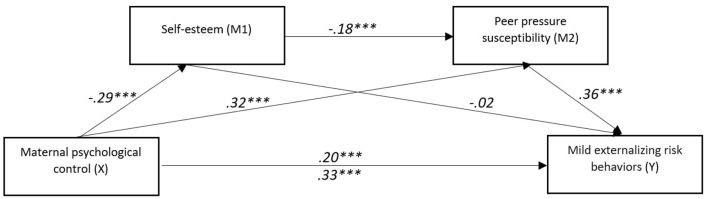
The mediating effect model of self-esteem and peer pressure susceptibility on the relationship between maternal psychological control and mild externalizing risk behaviors. ***p < 0.001.

In the third model, with mild externalizing risk behaviors as the outcome, the predictors explained 22% of the variance (*R*^2^ = 0.22, *F* = 27.50, *p* < 0.001). This effect is in the medium range (Cohen, 1988). Peer pressure susceptibility emerged as the strongest predictor, whereas self-esteem was not significant. Taken together, the findings suggest that the influence of maternal psychological control on externalizing risk behaviors operates through peer pressure susceptibility. Given the partial mediation and the explained variance of 22%, it is evident that additional factors beyond those tested here also play a role in this association. The total indirect effect of maternal psychological control on mild externalizing risk behaviors was significant, abcs = 0.14, 95% CI [0.08, 0.20]. Examination of the specific indirect effects showed that the pathway through peer pressure susceptibility was the strongest and significant [abcs = 0.11, 95% CI [0.07, 0.17]], followed by the sequential pathway through self-esteem and peer pressure susceptibility [abcs = 0.02, 95% CI [0.01, 0.04]]. The indirect effect via self-esteem alone was not significant [abcs = 0.01, 95% CI [−0.03, 0.03]]. These findings indicate that the effect of peer pressure susceptibility on mild externalizing risk behaviors is transmitted through peer pressure susceptibility, both directly and in sequence with self-esteem (Table 3; Figure 1).

**Table 3:** Multiple mediating effect analysis of maternal psychological control on adolescents' mild externalizing risk behaviors.

**Effect type**	**Effect**	**SE**	**Bootstrap 95% Cl**		**Effect ratio (%)**
			**Low**	**High**	
Total effect	0.037	0.005	0.026	0.047	100%
Direct effect	0.022	0.005	0.011	0.032	59.46%
**Mediation pathway**	**Effect**	**Boot SE**	**Bootstrap 95% Cl**		**Effect ratio (%)**
			**BootLow**	**BootHigh**	
Total indirect effect	0.015	0.003	0.009	0.022	40.54%
Path 1: Maternal psychological control → Self-esteem → Mild externalizing risk behaviors	0.001	0.002	−0.003	0.004	2.70%
Path 2: Maternal psychological control → Peer pressure susceptibility → Mild externalizing risk behaviors	0.013	0.003	0.008	0.019	35.14%
Path 3: Maternal psychological control → Self-esteem → Peer pressure susceptibility → Mild externalizing risk behaviors	0.002	0.001	0.001	0.004	5.41%

It can be concluded that the total indirect effect confirms the presence of significant mediation pathways. Peer pressure susceptibility independently mediates the relationship between maternal psychological control and mild externalizing risk behaviors. While self-esteem alone does not significantly mediate the relationship between maternal psychological control and undesirable normative behavior, maternal psychological control affects self-esteem, which in turn increases susceptibility to peer pressure, ultimately leading to an increase in mild externalizing risk behaviors.

The corresponding paternal results for this mediation are presented in Supplementary Tables S1, S2 and Supplementary Figure S1.

### Test of the mediating effects of self-esteem and peer pressure susceptibility between maternal emotional warmth and mild externalizing risk behaviors

**Figure 2:**
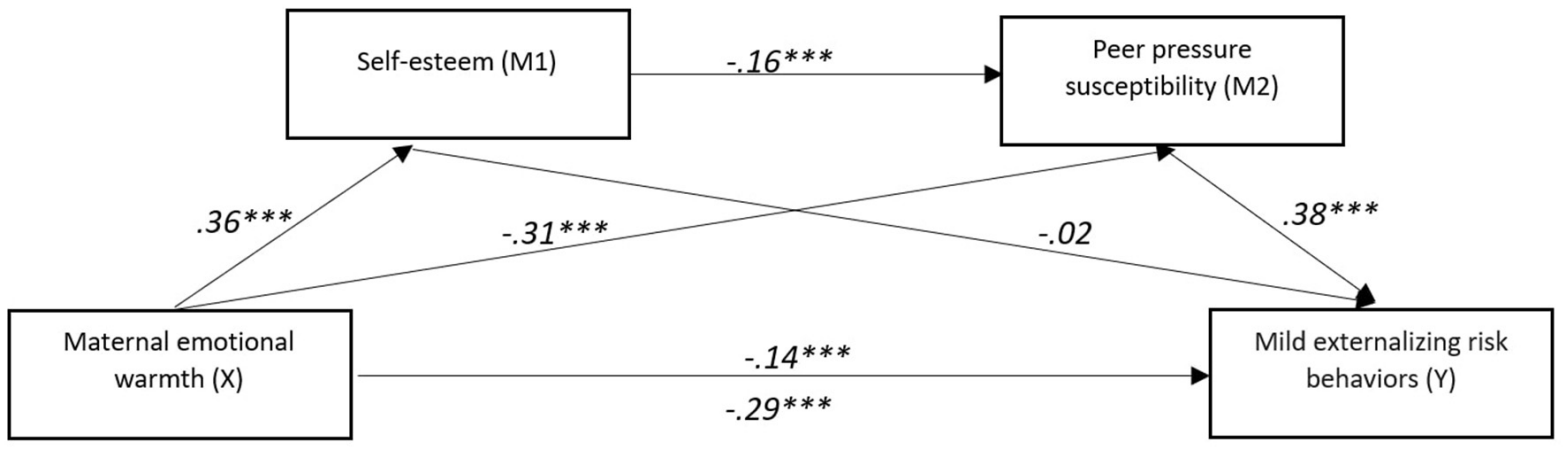
The mediating effect model of self-esteem and peer pressure susceptibility on the relationship between maternal emotional warmth and mild externalizing risk behaviors. **p < 0.01, ***p < 0.001.

Maternal emotional warmth has a significant positive effect on self-esteem (β = 0.36, *p* < 0.001), indicating that greater maternal emotional warmth is associated with higher levels of self-esteem (Table 4). Maternal emotional warmth (β = −0.31, *p* < 0.001) and self-esteem (β = −0.16, *p* < 0.01) have significant adverse effects on peer pressure susceptibility, suggesting that adolescents who receive higher maternal support and those with higher self-esteem are less susceptible to peer pressure. The direct impact of maternal emotional warmth on mild externalizing risk behaviors is significant and negative (β = −0.29, *p* < 0.0001) but weakens when mediators are included (β = −0.14, *p* < 0.01). While peer pressure susceptibility alone plays a significant role in this relation, self-esteem alone does not significantly affect mild externalizing risk behaviors; therefore, it could not be the single mediator. When included in the model relating maternal emotional warmth to mild externalizing risk behaviors alongside peer pressure susceptibility, the model becomes significant (Table 5, Figure 2).

**Table 4:** Regression model of the effect of maternal emotional warmth on adolescents' mild externalizing risk behaviors.

**Variables**	**β**	***t***	***p***	***R* ^2^**	***F***
**Step 1 outcome variable: self-esteem**					
Control variable: Gender	−0.24	−5.23	0.00	0.21	53.470^*^
Predictor: Emotional warmth	0.36	7.94	0.00		
**Step 2 outcome variable: peer pressure susceptibility**					
Control variable: Gender	0.03	0.66	0.51	0.17	26.108^*^
Predictor: Emotional warmth	−0.31	−6.11	0.00		
Mediator 1: Self-esteem	−0.16	−3.06	0.00		
**Step 3 outcome variable: mild externalizing risk behaviors**					
Control variable: Gender	−0.05	−1.11	0.27	0.20	25.132^*^
Predictor: Emotional warmth	−0.14	−2.75	0.01		
Mediator 1: Self-esteem	−0.02	−0.35	0.73		
Mediator 2: Peer pressure susceptibility	0.38	7.65	0.00		

^*^p < 0.001.

**Table 5:** Multiple mediating effect analysis of maternal emotional warmth on adolescents' mild externalizing risk behaviors.

**Effect type**	**Effect**	**SE**	**Bootstrap 95% Cl**		**Effect ratio (%)**
			**Low**	**High**	
Total effect	−0.024	0.004	−0.032	−0.016	100%
Direct effect	−0.012	0.004	−0.021	−0.003	50%
**Mediation pathway**	**Effect**	**Boot SE**	**Bootstrap 95% Cl**		**Effect ratio (%)**
			**BootLow**	**BootHigh**	
Total indirect effect	−0.012	0.002	−0.018	−0.007	50%
Path 1: Maternal emotional warmth → Self-esteem → Mild externalizing risk behaviors	−0.001	0.002	−0.004	0.003	4.17%
Path 2: Maternal emotional warmth → Peer pressure susceptibility → Mild externalizing risk behaviors	−0.010	0.002	−0.015	−0.006	41.67%
Path 3: Maternal emotional warmth → Self-esteem → Peer pressure susceptibility → Mild externalizing risk behaviors	−0.002	0.001	−0.004	−0.0003	8.33%

The second mediating model examined the relationship between maternal emotional warmth and mild externalizing risk behaviors, mediated by self-esteem and peer pressure susceptibility.

In the first model (Table 4), predictors explained 21% of the variance in self-esteem (*R*^2^= 0.21, *F* = 63.47, *p* < 0.001), indicating a medium effect size (Cohen, 1988). Both gender and maternal emotional warmth were significant, with emotional warmth showing a more substantial effect on self-esteem.

In the second model, predictors accounted for 17% of the variance in peer pressure susceptibility (*R*^2^= 0.17, *F* = 26.11, *p* < 0.001), again a medium effect (Cohen, 1988). Maternal emotional warmth was positively associated with susceptibility to peer pressure, while self-esteem contributed negatively and partially mediated this relationship.

In the third model, predictors explained 20% of the variance in mild externalizing risk behaviors (*R*^2^= 0.20, *F* = 25.13, *p* < 0.001), also a medium effect (Cohen, 1988). Peer pressure susceptibility was the strongest predictor, while self-esteem was not significant.

The analysis revealed a significant total indirect effect of maternal emotional warmth on mild externalizing risk behaviors, abcs = −0.15, 95% CI [−0.21, −0.09]. Among the specific pathways, the indirect effect through peer pressure susceptibility was the strongest and significant, abcs = −0.12, 95% CI [−0.17, −0.07]. A more minor but still significant sequential effect was observed through self-esteem and peer pressure susceptibility, abcs = −0.02, 95% CI [−0.04, −0.01]. In contrast, the indirect effect through self-esteem alone was not significant, abcs = −0.01, 95% CI [−0.05,0.03]. These findings suggest that the impact of maternal emotional warmth on mild externalizing risk behaviors is primarily transmitted through peer pressure susceptibility, with self-esteem contributing only when operating in sequence with peer pressure susceptibility.

The corresponding paternal results for this mediation are presented in Supplementary Tables S3, S4 and Supplementary Figure S2.

### Test of the mediating effects of self-esteem and peer pressure susceptibility between maternal psychological control and internalizing risk behaviors

In the third mediation (Table 6), we examined the role of self-esteem and peer pressure as mediators in the relationship between perceived maternal psychological control and internalizing risk behaviors (suicidal and self-aggressive behaviors). Maternal psychological control has a significant adverse effect on self-esteem (β = −0.29, *p* < 0.001) and a significant positive effect on peer pressure (β = 0.31, *p* < 0.001). Higher maternal control is associated with lower self-esteem and is linked to increased susceptibility to peer pressure. As noted before, self-esteem has a significant adverse effect on peer pressure (β = −0.18, *p* < 0.001), suggesting that individuals with higher self-esteem are less susceptible to peer pressure. Self-esteem has a significant negative effect on internalizing risk behaviors (β = −0.30, *p* < 0.001), and peer pressure susceptibility has a significant positive effect on internalizing risk behaviors (β = 0.20, *p* < 0.001). Considering the model with mediation, maternal psychological control has a significant direct effect on internalizing risk behaviors (β = 0.19, *p* < 0.001), but this effect is reduced when self-esteem and peer pressure susceptibility are included as mediators.

**Table 6:** Regression model of the effect of maternal psychological control on adolescents' internalizing risk behaviors.

**Variables**	**β**	***t***	***p***	***R* ^2^**	***F***
**Step 1 outcome variable: self-esteem**					
Control variable: Gender	−0.26	−5.59	0.00	0.17	40.853^*^
Predictor: Psychological control	−0.29	−6.27	0.00		
**Step 2 outcome variable: peer pressure susceptibility**					
Control variable: Gender	0.03	0.599	0.55	0.17	27.064^*^
Predictor: Psychological control	0.31	6.41	0.00		
Mediator 1: Self-esteem	−0.18	−3.51	0.00		
**Step 3 outcome variable: internalizing risk behaviors**					
Control variable: Gender	0.11	2.68	0.01	0.31	44.721^*^
Predictor: Psychological control	0.19	4.11	0.00		
Mediator 1: Self-esteem	−0.30	−6.40	0.00		
Mediator 2: Peer pressure susceptibility	0.25	4.33	0.00		

^*^p < 0.001.

In the first model, with self-esteem as the outcome, psychological control and gender together explained 17% of the variance (*R*^2^ = 0.17, *F* = 40.85, *p* < 0.001), which represents a medium effect size. Psychological control was a significant negative predictor, indicating that higher levels of maternal psychological control are associated with lower adolescent self-esteem.

In the second model, with peer pressure susceptibility as the outcome, the predictors again explained 17% of the variance (*R*^2^ = 0.17, *F* = 27.06, *p* < 0.001). Psychological control was a positive predictor of susceptibility to peer pressure, while self-esteem was a significant negative predictor. This suggests that adolescents with lower self-esteem are more vulnerable to peer influence.

In the third model, with internalizing risk behaviors as the outcome, the predictors accounted for 31% of the variance (*R*^2^ = 0.31, *F* = 44.72, *p* < 0.001), which indicates a large effect size. Psychological control, self-esteem, and peer pressure susceptibility were all significant predictors. Notably, self-esteem showed the strongest negative association, followed by peer pressure susceptibility as a positive predictor. This pattern suggests that the pathway from psychological control to internalizing risk behaviors is transmitted through reduced self-esteem, as well as increased susceptibility to peer influence.

Analyses of indirect effects further support these findings. The total indirect effect of psychological control on internalizing risk behaviors was significant [abcs = 0.16, 95% CI [0.10, 0.22]], reflecting a medium effect size (Cohen, 1988). The strongest indirect pathway was via self-esteem [abcs = 0.09, 95% CI [0.05, 0.13]], followed by the path via peer pressure susceptibility [abcs = 0.06, 95% CI [0.02, 0.11]]. A more minor but significant sequential indirect effect was also observed through both self-esteem and peer pressure susceptibility [abcs = 0.01, 95% CI [0.00, 0.02]].

Taken together, these results indicate that maternal psychological control contributes to higher levels of internalizing risk behaviors among adolescents, primarily through its detrimental impact on self-esteem and, to a lesser degree, through heightened susceptibility to peer pressure. The relatively large proportion of explained variance (31%) suggests that these mediating processes are meaningful, although additional factors beyond those included in this model are also likely to play a role.

**Figure 3:**
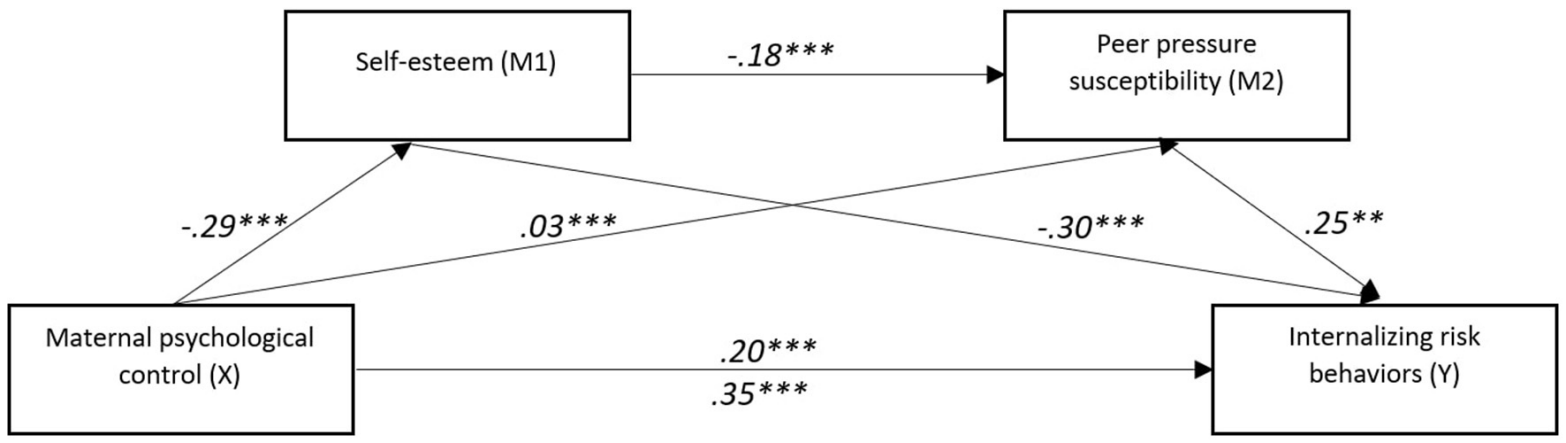
The mediating effect model of self-esteem and peer pressure susceptibility on the relationship between maternal psychological control and internalizing risk behaviors. **p < 0.01, ***p < 0.001.

This suggests that mediation processes contribute to explaining this relationship. As shown in Table 7 and Figure 3, the total indirect effect, representing the sum of all mediation pathways, was also significant, confirming the presence of mediation effects. The most potent mediation effect occurs through self-esteem alone, indicating that maternal psychological control reduces self-esteem, which in turn increases tendencies toward internalizing risk behaviors. Peer pressure susceptibility also serves as a significant mediator, both directly and in serial mediation with self-esteem. The weakest mediation effect is observed in the serial pathway, suggesting that while self-esteem influences peer pressure, its impact on internalizing risk behaviors is more direct.

**Table 7:** Multiple mediating effect analysis of maternal psychological control on adolescents' internalizing risk behaviors.

**Effect type**	**Effect**	**SE**	**Bootstrap 95% Cl**		**Effect ratio (%)**
			**Low**	**High**	
Total effect	0.048	0.006	0.036	0.061	100%
Direct effect	0.026	0.006	0.014	0.039	54.17%
**Mediation pathway**	**Effect**	**Boot SE**	**Bootstrap 95% Cl**		**Effect ratio (%)**
			**BootLow**	**BootHigh**	
Total indirect effect	0.022	0.005	0.014	0.031	45.83%
Path 1: Maternal psychological control → Self-esteem → Internalizing risk behaviors	0.012	0.003	0.007	0.019	25%
Path 2: Maternal psychological control → Peer pressure susceptibility → Internalizing risk behaviors	0.009	0.003	0.007	0.019	18.75%
Path 3: Maternal psychological control → Self-esteem → Peer pressure susceptibility → Internalizing risk behaviors	0.001	0.001	0.0003	0.003	2.08%

The corresponding paternal results for this mediation are presented in Supplementary Tables S5, S6 and Supplementary Figure S3.

### Test of the mediating effects of self-esteem and peer pressure susceptibility between maternal emotional warmth and internalizing risk behaviors

In the fourth analysis, we examined the mediating effect of self-esteem and peer pressure susceptibility on the relationship between maternal emotional warmth and internalizing risk behaviors. Maternal emotional warmth was found to be a significant positive predictor of self-esteem (β = 0.36, *p* < 0.001), i.e., greater maternal emotional support is associated with higher levels of self-esteem (Table 8). Maternal emotional warmth was found to negatively predict susceptibility to peer pressure (β = −0.30, *p* < 0.001), suggesting that higher levels of maternal emotional warmth are associated with reduced susceptibility to peer pressure. Maternal emotional warmth has a negative effect on internalizing risk behaviors (β = −0.28, *p* < 0.001). Similarly, self-esteem was found to have a negative impact on internalizing risk behaviors (β = −0.26, *p* < 0.001), suggesting that adolescents with higher self-esteem have lower tendencies toward internalizing risk behaviors. Conversely, peer pressure susceptibility positively predicted internalizing risk behaviors (β = 0.18, *p* < 0.001), indicating that higher levels of peer pressure are associated with higher levels of internalizing risk behaviors.

**Table 8:** Regression model of the effect of maternal emotional warmth on adolescents' internalizing risk behaviors.

**Variables**	**β**	***t***	***p***	***R* ^2^**	***F***
**Step 1 outcome variable: self-esteem**					
Control variable: Gender	−0.24	−5.30	00	0.22	53.989^*^
Predictor: emotional warmth	0.36	7.96	00		
**Step 2 outcome variable: peer pressure susceptibility**					
Control variable: Gender	0.03	0.57	0.57	0.16	24.964^*^
Predictor: Emotional warmth	−0.30	−5.95	0.00		
Mediator 1: Self-esteem	−0.16	−3.05	0.00		
**Step 3 outcome variable: internalizing risk behaviors**					
Control variable: Gender	0.11	2.64	0.01	0.35	51.428^*^
Predictor: Emotional warmth	−0.28	−6.01	0.00		
Mediator 1: Self-esteem	−0.26	−5.48	0.00		
Mediator 2: Peer pressure susceptibility	0.18	4.03	0.00		

^*^p < 0.001.

**Figure 4:**
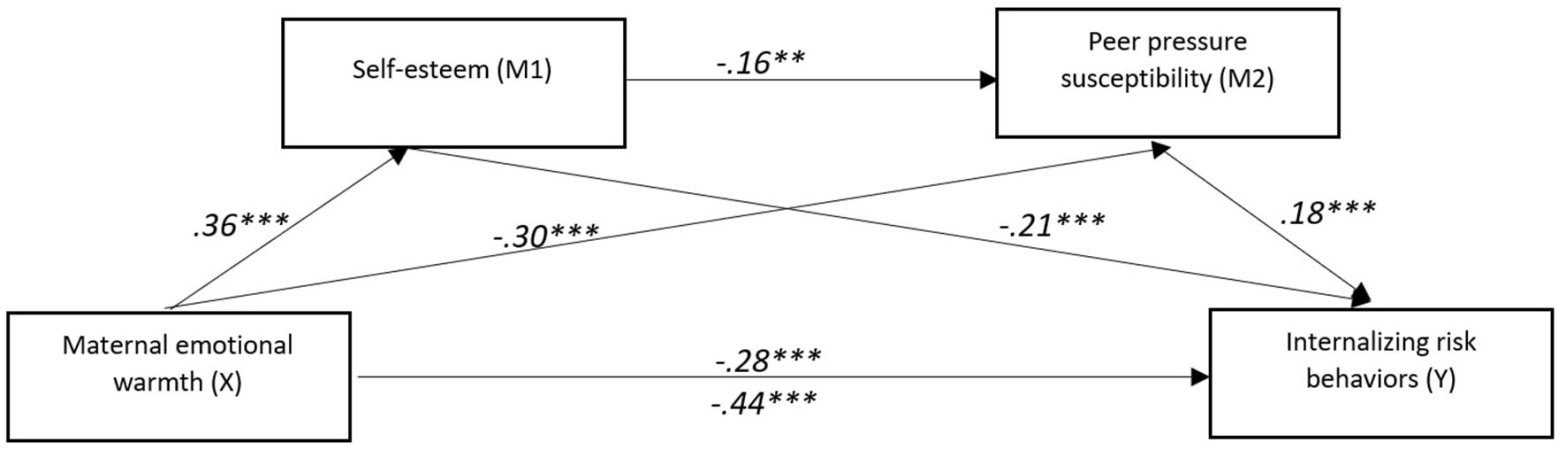
The mediating effect model of self-esteem and peer pressure susceptibility on the relationship between maternal emotional warmth and internalizing risk behaviors. **p < 0.01, ***p < 0.001.

In the first model (Table 8, Table 9 and Figure 4), with self-esteem as the outcome, emotional warmth and gender explained 22% of the variance (*R*^2^ = 0.22, *F* = 53.99, *p* < 0.001), which corresponds to a medium-to-large effect (Cohen, 1988). Emotional warmth was a strong positive predictor, indicating that adolescents who perceive higher levels of maternal emotional warmth also report higher self-esteem.

**Table 9:** Multiple mediating effect analysis of maternal emotional warmth on adolescents' internalizing risk behaviors.

**Effect type**	**Effect**	**SE**	**Bootstrap 95% Cl**		**Effect ratio (%)**
			**Low**	**High**	
Total effect	−0.046	0.005	−0.055	−0.037	100%
Direct effect	−0.030	0.005	−0.020	−0.055	65.22%
**Mediation pathway**	**Effect**	**Boot SE**	**Bootstrap 95% Cl**		**Effect ratio (%)**
			**BootLow**	**BootHigh**	
Total indirect effect	−0.017	0.003	−0.024	−0.011	36.96%
Path 1: Maternal emotional warmth → Self-esteem → Internalizing risk behaviors	−0.010	0.003	−0.015	−0.005	21.74%
Path 2: Maternal emotional warmth → Peer pressure susceptibility → Internalizing risk behaviors	−0.006	0.002	−0.011	−0.001	13.04%
Path 3: Maternal emotional warmth → Self-esteem → Peer pressure susceptibility → Internalizing risk behaviors	−0.001	0.001	−0.002	−0.0002	2.17%

In the second model, predicting peer pressure susceptibility, the predictors explained 16% of the variance (*R*^2^ = 0.16, *F* = 24.96, *p* < 0.001), indicating a medium effect. Emotional warmth was negatively related to susceptibility, while self-esteem also contributed as a negative predictor, showing that both higher warmth and higher self-esteem are associated with reduced vulnerability to peer influence.

In the third and final model, with internalizing risk behaviors as the dependent variable, the predictors explained 35% of the variance (*R*^2^ = 0.35, *F* = 51.43, *p* < 0.001), indicating a large effect size. Emotional warmth was found to have a direct negative association with internalizing risk behaviors, and both self-esteem and peer pressure susceptibility were identified as significant mediators in this association.

Analyses of indirect effects confirmed that the total indirect effect of emotional warmth on internalizing risk behaviors was significant [abcs = −0.16, 95% CI [−0.22, −0.10]]. The strongest pathway was through self-esteem [abcs = −0.09, 95% CI [−0.14, −0.05]], followed by the path through peer pressure susceptibility [abcs = −0.05, 95% CI [−0.10, −0.01]]. A more minor but significant sequential indirect effect was also detected, operating first through self-esteem and then through peer pressure susceptibility [abcs = −0.01, 95% CI [−0.02, −0.00]]. The contrasts between indirect effects further indicated that the pathway via self-esteem was significantly stronger than both the pathway via peer pressure susceptibility and the sequential path. In addition, the pathway via peer pressure susceptibility alone was stronger than the sequential pathway.

Overall, these findings suggest that maternal emotional warmth reduces internalizing risk behaviors primarily by fostering higher self-esteem in adolescents, while reduced susceptibility to peer pressure plays a more minor but meaningful role. The presence of both direct and indirect effects suggests that emotional warmth serves as a robust protective factor, although other unmeasured influences may also contribute to the development of internalizing problems.

The corresponding paternal results for this mediation are presented in Supplementary Tables S7, S8 and Supplementary Figure S4.

## Discussion

A series of hierarchical regression analyses examined the serial mediation of self-esteem and peer pressure susceptibility as mediators in the relationship between both maternal and paternal psychological control and emotional warmth and the emergence of mild externalizing and internalizing risk behaviors during early adolescence. Eight serial mediation models were identified (four sets of maternal results are presented in the main text, while the corresponding four sets of paternal results are provided in Supplementary material).

### The influence of parental psychological control and emotional warmth on adolescents' externalizing risk behaviors: the direct effect and indirect effect

Adolescence is a critical developmental period marked by heightened sensitivity to environmental influences, particularly those of parents and peers. Parental psychological control and emotional warmth are key factors shaping adolescents' behavioral outcomes. Our data indicate that parental psychological control has both direct and indirect effects (via peer susceptibility) on the engagement of young adolescents in mild externalizing risk behaviors. Prior research also indicated that higher levels of perceived parental psychological control are associated with increased engagement in risk and delinquent behaviors among adolescents (Klarin and Ãerda, 2014; Pettit et al., 2001; Smetana and Daddis, 2002). In this study, we examined some of the mechanisms through which this relationship operates, specifically whether parental influence affects the development of self-esteem and whether susceptibility to peer pressure acts as a mediating factor in this process. We hypothesized that self-esteem and susceptibility to peer pressure mediate the relationship between parental psychological control and mild externalizing risk behaviors. A positive parent-child relationship is expected to influence the development of self-esteem, which in turn may affect the likelihood of submitting to peer influence, which may then influence the probability of engaging in externalizing risk behaviors.

The study confirmed a negative association between parental psychological control and self-esteem, consistent with prior research (e.g., Kim et al., 2017; Shek, 2007). Specifically, higher levels of parental psychological control were linked to lower self-esteem. Psychological control, characterized by manipulative behaviors and passive aggression (Silk et al., 2003), as well as intrusive, manipulative, and coercive behavior, negatively affects a child's development (Silk et al., 2003). Such forms of parental behavior can be unfavorable to children's psychosocial development; therefore, they are understandably linked to reduced adolescent self-esteem. Considering the age of the participants—early adolescence, a period when self-esteem is fragile and unstable—these findings are particularly significant, as they highlight the vulnerability of young adolescents to external influences and the potential long-term impact on their psychological and social development. Furthermore, the study revealed a significant negative association between self-esteem and susceptibility to peer pressure, supporting the findings of previous research (e.g., Borra, 2024). Specifically, adolescents with lower self-esteem were found to be more prone to peer influence. Additionally, parental psychological control was also identified as a contributing factor, increasing adolescents' vulnerability to peer pressure. These results underscore the complex interplay between internal self-perceptions and familial influences in shaping adolescents' responses to social pressures.

Early adolescence is marked by a heightened vulnerability to peer pressure (Lebedina-Manzoni and Ricijaš, 2013). During this developmental stage, the influence of peers on an individual is more significant than at any other life phase (Berndt, 1979; Lebedina-Manzoni and Ricijaš, 2013). This increased susceptibility arises from a strong desire for acceptance within peer groups, prompting younger adolescents to often conform to their peers, which may lead them to overlook their preferences and beliefs (Lebedina-Manzoni and Ricijaš, 2013). Within this framework, peer interactions have a significant impact on adolescents' decisions to engage in risky and delinquent behaviors (Abbey et al., 2006; Blakemore and Mills, 2014; Miller et al., 2009). It is possible that in the present study, peer influence played an even stronger role than might typically be observed in highly individualistic societies. Croatia, while not a strictly collectivistic culture, cannot be described as purely individualistic either. In such a sociocultural setting, belonging to a peer group and maintaining group harmony may carry particular importance. The wellbeing of the group, and even the willingness to sacrifice individual interests for the sake of the group, can at times outweigh personal goals.

In adolescence, where peer acceptance is a central developmental task, the fear of exclusion may be especially pronounced. Being excluded from the group could be experienced as a loss of security, support, and connectedness. Under such circumstances, adolescents may be more likely to engage in risk behaviors when pressured by peers, not necessarily out of personal inclination, but to maintain their membership and sense of belonging. Consistent with this assumption are the findings of Šimić Šašić and Klarin (2009), who reported that as many as 92.7% of high school students in Croatia had engaged in some form of cheating at least once during knowledge assessments. The most widely accepted form was passive cheating, that is, helping other students during examinations. The authors emphasized that such behavior is often not perceived as cheating at all, but rather interpreted as an altruistic act. Among students, it is broadly accepted as appropriate or even socially expected behavior within peer groups.

We have established that peer pressure susceptibility plays a significant mediating role between parental psychological control and risk behaviors, both independently and in combination with self-esteem. According to the data in this research, self-esteem alone does not directly predict mild externalizing risk behaviors; rather, it affects susceptibility to peer pressure, making it an indirect vulnerability factor. Although the initial correlation analysis revealed a weak association between self-esteem and mild externalizing risk behaviors, this direct relationship disappeared in the regression model, likely due to the inclusion of other variables. These results are somewhat unexpected, given that numerous prior studies (e.g., Dobešová Cakirpaloglu et al., 2020; Modecki and Uink, 2018) have identified self-esteem as a contributing factor to the emergence of externalizing risk behaviors. Individuals with low self-esteem struggle to maintain a positive self-image (Leary, 1999; Zimmerman et al., 1997), and as a result, they may seek to establish or increase their self-esteem by engaging in risk behaviors that are preferred within a peer group of delinquents. Adolescents with low self-esteem often have negative self-perceptions and difficulty recognizing their value, making them more vulnerable to peer pressure as they seek acceptance and a sense of belonging. Furthermore, low self-esteem has been linked to the development of delinquency (Harter, 1990; Hirsch and DuBois, 1991), academic failure, and social isolation (Cruz et al., 2023; Szcześniak et al., 2020), all of which may further contribute to vulnerability to peer pressure.

The weak correlation identified in this study may be associated with several reasons. One potential reason is the developmental stage of the sample; younger adolescents may not exhibit sufficiently varied levels of externalizing risk behaviors for these relationships to be evident. Additionally, the influence of other factors, such as peer pressure, family environment, or cultural context, may have played a more significant role in this age group, thereby diminishing the predictive power of self-esteem alone.

The study also demonstrated that self-esteem plays an important role in reducing adolescents' susceptibility to peer pressure. Higher levels of self-esteem were associated with decreased influence of peers on adolescents' behavior. This finding aligns with previous research, which has consistently shown that increased self-esteem serves as a protective factor against external influences, including peer pressure (Omisola et al., 2022) and engagement in externalizing risk behaviors (Martínez-Casanova et al., 2024; Rosenberg et al., 1989).

Additionally, parental emotional warmth—characterized by support and affection—was found to have a positive influence on self-esteem. In turn, this elevated self-esteem contributed to a diminished likelihood of adolescents succumbing to peer pressure. The research also confirmed that parental love and support directly enhance adolescents' self-esteem. The relationship between parental warmth and susceptibility to peer pressure has been established in prior studies (i.e., Chan and Chan, 2013), supporting the current findings. Similarly, the association between parental warmth and adolescents' self-esteem has also been confirmed in earlier research (i.e., Khaleque, 2017; Krauss et al., 2020; Wu et al., 2022), emphasizing the consistent role of parental emotional support in adolescent development. Self-esteem is not directly associated with externalizing risk behaviors, and therefore it is expected that, in the present model, its association with maternal warmth is not central. Instead, the relationship between maternal warmth and externalizing risk behaviors is better explained through the mediating role of susceptibility to peer pressure.

Analyzing the results related to mild externalizing risk behaviors, the influence of parental behavior—specifically emotional warmth and psychological control—on engagement in externalizing behaviors can be partially explained by susceptibility to peer influence and, to a lesser extent, by susceptibility combined with self-esteem. Since the study did not establish complete mediation but only a partial one, other factors not examined in this research may also contribute to this relationship. Such a finding is not uncommon, given that involvement in risk behaviors is a complex phenomenon influenced by a multitude of factors, including personal, familial, social, and cultural variables, all of which can affect the likelihood of engaging in risk behaviors. The analysis of the two mediator models concerning external mild risk behaviors reveals that models where peer pressure susceptibility acts as the sole mediator show significantly greater strength than the models that include both self-esteem and peer pressure susceptibility as mediators. This suggests that peer pressure susceptibility is a more influential factor in explaining the relationship between the independent variable and mild externalizing risk behaviors. In essence, while self-esteem may play a role, direct susceptibility to peer influence captures how these variables interact more effectively, indicating that peer dynamics have a more direct impact on adolescents than their self-perception. This highlights the critical importance of peer relationships in mild externalizing risk behaviors among young individuals.

### The influence of parental psychological control and emotional warmth on adolescents' internalizing risk behaviors: the direct effect and indirect effect

Having examined the role of parenting behavior in relation to mild externalizing risk behaviors, where susceptibility to peer pressure was identified as the more significant mediator, the focus now shifts to internalizing risk behaviors.

The findings of the present study indicate that maternal psychological control is a positive predictor of internalizing risk behaviors in early adolescence. Previous research has consistently demonstrated that parental psychological control represents a significant predictor of internalizing difficulties, including symptoms of depression (Barber, 1996). Furthermore, findings by Symeou and Georgiou (2017) provide additional support for the association between parental psychological control and internalizing risk behaviors in adolescence. Self-injury, as a form of internalizing risk behaviors, indicates that an intrusive caregiving environment contributes to the formation of negative self and other representations, which further elevates the risk of non-suicidal self-injury (NSSI) through various motivational and attitudinal factors (Yates, 2004). Previous research has also shown that parenting practices and characteristics can affect adolescent NSSI by influencing unmet psychological needs and emotional issues (e.g., Guo et al., 2022; Huang et al., 2022). Additionally, pre-adolescents (12-year-olds) who practiced non-suicidal self-injury (NSSI) exhibited greater parental psychological control compared to their peers who did not engage in self-injury (Baetens et al., 2014).

Therefore, in light of the above, it can be concluded that the findings of this study are consistent with those of previous research. Conversely, parental emotional support, i.e., warmth, has a protective effect, as it positively influences self-esteem and decreases susceptibility to peer pressure, thereby reducing the risk of negative behaviors.

The influence of family factors, as well as the quality of parent-child relationships, on the emergence of internalizing risk behaviors has been substantiated by previous research (e.g., Fortune et al., 2016; Tschan et al., 2015). The significance of parental emotional support and warmth is further corroborated by (Aguilar-Yamuza et al. 2023), who found that children with internalizing problems often have parents who fail to provide adequate support. Additionally, parental empathy, emotional warmth, and sensitivity to the child's needs are associated with the development of effective emotional regulation in children (Kliewer et al., 1996). Consequently, it can be presumed that parental emotional warmth is a crucial factor in the healthy emotional development of children. Research focusing specifically on mothers further underscores this point, showing that supportive maternal responses to children's emotions are linked to a reduced likelihood and lower frequency of NSSI among adolescent girls (White et al., 2021). In contrast, unsupportive responses are associated with more severe NSSI, even after accounting for depression and other risk factors (White et al., 2021). Moreover, evidence indicates that maternal emotional warmth functions as a protective factor (Zhang et al., 2024a,b), reducing the risk and severity of internalizing problem behaviors in adolescence (Rothenberg et al., 2020). In the Croatian context, where adolescents typically maintain closer relationships with their mothers than with their fathers, the lack of maternal warmth and support from this primary caregiver may have a particularly adverse effect on self-esteem and the emergence and development of internalizing risk behaviors. As Hazan and Shaver (1994) argue, children tend to form stronger bonds with attachment figures who are more consistently available to them, which further explains why maternal influence is especially pronounced.

When considering parental roles more broadly, previous research (e.g., Basili et al., 2021) indicates that maternal and paternal psychological control may have distinct effects on adolescent adjustment. Specifically, maternal psychological control has been linked to higher levels of antisocial behavior in adolescents, whereas paternal psychological control has been associated with lower levels of anxious–depressed symptoms. In contrast to these findings, the present study showed that both maternal and paternal psychological control were related to mild externalizing risk behaviors as well as to internalizing risk behaviors.

A noteworthy finding is that susceptibility to peer pressure is a statistically significant predictor of internalizing risk behaviors. These results suggest a distinct relationship between peer pressure and internalizing risk behaviors in youth. It can be hypothesized that some participants engaged in self-harm due to or under peer pressure. Previous research has confirmed the link between peer pressure and self-injury (e.g., James, 2013; Prinstein et al., 2010). Prinstein et al. (2010) determined that peer self-injury during adolescence is associated with individual self-injury, revealing that friends' self-harm is a more significant predictor of self-injury than depressive symptoms. A concerning finding by (Tørmoen et al. 2020) examined trends in adolescent self-injury from 2002 to 2018, discovering an increase in self-injury prevalence from 4.1% in the initial measurement (2002) to 16.2% in the later measurement (2018). This rise was relatively more pronounced in younger adolescents than in older ones. While adolescent self-injury is often associated with coping with distressing emotional states (e.g., anger and depression) and various externalizing and internalizing behavioral problems (Peterson et al., 2008), recent literature also examines it as a trend influenced by peers. Heilbron and Prinstein (2008) highlight that peers are a source of information about self-injury and may normalize such behaviors in stressful situations. In such an environment, self-injury may become a potential emotion regulation strategy for young individuals struggling to manage negative emotions. If such emotion regulation is linked with peers perceived as role models (popular peers, close friends, or group members with whom they identify), self-injurious behavior not only aids in coping with negative emotions but also contributes to achieving a desired self-image. Peers may influence the methods of self-injury individuals choose, often opting for behaviors similar to those of their peers (e.g., burns and cuts) and engaging in more severe forms of such behavior to demonstrate extreme forms of behavior, thereby projecting a rebellious identity (Heilbron and Prinstein, 2008).

Furthermore, self-esteem emerges as a statistically significant negative predictor of internalizing risk behaviors. Such findings are anticipated, given that numerous prior studies (e.g., Cvetković et al., 2023; Martínez-Casanova et al., 2024; Mullan et al., 2023) have identified low self-esteem as a risk factor for depressive symptoms, suicidal ideation, and anxiety. Orth et al. (2008), in a longitudinal study encompassing participants aged 15 to 21 years, identified low self-esteem as a stronger predictor of depression than depression predicting low self-esteem. In the present study, we established that self-esteem plays a crucial protective role and is an essential mechanism in shaping adolescent behavior. Parental emotional support can influence internalizing risk behaviors both directly and indirectly through its impact on self-esteem and susceptibility to peer pressure.

Although the model in which parental behavior influences the emergence of internalizing risk behaviors is mediated by both self-esteem and susceptibility to peer pressure, the data indicate that a more parsimonious model with self-esteem as the sole mediator provides a better fit. These results support the notion that parental support exerts its protective effect on adolescents more through its impact on self-esteem than through influencing susceptibility to peer pressure (or that self-esteem, indirectly via susceptibility to peer pressure, affects internalizing risk behaviors). Consequently, the findings suggest that fostering parental warmth and support can strengthen adolescents' self-esteem, thereby reducing the risk of internalizing risk behaviors.

The finding that internalizing behaviors were more strongly associated with self-esteem than with susceptibility to peer pressure may be understood in light of the cultural characteristics of Croatian society. Croatia, while not fully collectivistic, is often described as a culture where family bonds remain intense and emotional closeness within the household is highly valued. In such a context, the family—particularly maternal warmth or control—can play a central role in shaping adolescents' sense of self-worth. Since internalizing problems such as anxiety or withdrawal are closely tied to self-perceptions, it is plausible that adolescents' self-esteem, mainly developed within the family environment, is a stronger predictor than peer dynamics. In accordance with these findings, (Jokić and Ristić Dedić 2023) found, in their large-scale research involving 12,000 Croatian youth, that the vast majority of Croatian youth express high satisfaction and trust in their relationships with their parents, highlighting the strength of family bonds. Family was consistently described as the most influential factor in the development and wellbeing of children and adolescents in Croatia, with youth reporting closer relationships with parents compared to peers. Furthermore, family is recognized as the most influential factor in the developmental outcomes of children and adolescents.

It is particularly significant to conclude that self-esteem is an important mediator in the relationship between parental behavior and internalizing risk behaviors. Conversely, peer influence primarily mediates the relationship between parental behavior and externalizing risk behaviors. These findings underscore the crucial role of self-esteem in buffering against internalizing risk behaviors associated with parental influences, while peer pressure appears to be more relevant for externalizing risk behaviors.

Our research demonstrated that although inadequate parenting (characterized by high psychological control and/or low emotional warmth) contributes to the emergence of risk behaviors (both internalizing and externalizing), part of this relationship can be explained by the effects of self-esteem and susceptibility to peer influence. It is possible that both maternal and paternal psychological control and warmth (in some part) can lead to lower self-esteem, making younger adolescents more susceptible to peer pressure, which in turn can increase their desire to enhance their self-esteem and heighten the likelihood of engaging in risk behaviors.

In Croatia, where family ties are often intense and close, parents may have a particularly important influence on adolescents' internal experiences, such as anxiety or low mood. At the same time, externalizing behaviors, such as aggression or rule-breaking, appear to be more influenced by peers, reflecting the role of social groups outside the family.

These findings suggest that in cultural settings with strong family connections, maternal behaviors may be significant for internalizing outcomes, while peer influence plays a larger role for externalizing behaviors. This highlights the combined impact of family and social context on adolescent development.

This study contributes to the field of developmental psychology by shedding light on the relationship between various aspects of parenting and adolescents' adjustment. It emphasizes the importance of both individual and social factors—such as self-esteem, psychological control, emotional warmth, and peer pressure—in understanding what might lead younger adolescents toward risk-taking behavior. The results also suggest that how parenting influences externalizing behaviors differs from the pathways that lead to internalizing ones. Altogether, these findings provide a useful starting point for future research and can inform more targeted efforts to support young people's emotional and behavioral development.

## Implications

This research has several notable contributions and implications.

The study was conducted in a cultural context characterized by the transitional nature of post-socialist societies, which combine elements of both collectivism and individualism. Croatia, as a post-socialist country, has moved toward Western ideals of independence and self-realization, yet many aspects of family life and peer relations still reflect older patterns of interdependence. Parents often emphasize security and loyalty, while schools and the broader social environment increasingly promote autonomy, confidence, and personal growth. This cultural context can create a mixed experience because close family ties and expectations of conformity remain strong, while peers, media, school, and other influences encourage individuals to develop self-esteem, assert themselves among peers, and take initiative. Peer groups thus become an essential space where they learn how to balance belonging with independence. This gap between old and new cultural orientations makes post-socialist countries, such as Croatia, a valuable context for psychological research as they show how shifts in social structure and values shape young people's identity, motivation, and sense of self.

The findings of this study highlight the complex interplay between parental emotional warmth, parental psychological control, self-esteem, peer influence, and the development of risk behaviors in early adolescence. Specifically, the results demonstrate how parental psychological control and warmth influence adolescents' susceptibility to peer pressure and their self-esteem, which in turn partially mediate the emergence of internalizing and externalizing risk behaviors. By confirming these distinct pathways, the study advances theoretical models of adolescent development and provides empirical support for targeted intervention strategies. Furthermore, the study has demonstrated that different mediating mechanisms may be relevant depending on the type of risk behaviors. Specifically, while susceptibility to peer pressure (measured concurrently with self-esteem) was identified as a more significant mediator in the emergence of externalizing risk behaviors, self-esteem proved to be a more critical mediator—alongside peer pressure—in the case of internalizing risk behaviors. This finding underscores the necessity of distinguishing between various forms of adolescent risk behaviors when examining the mediating roles of personal and social factors.

Another significant contribution of this study concerns the coverage of the sample and population. The research was conducted with younger adolescents and included the entire population of eighth-grade students in a city of about 76,000 inhabitants. Although 62% of the students completed the questionnaire, all had the opportunity to participate. This approach enhances the representativeness of the sample, making the findings more generalizable to the broader population. Focusing on younger adolescents is especially valuable, as this developmental period is critical for the formation of self-esteem, susceptibility to peer pressure, and the onset of risk behaviors. The results, therefore, provide important insights into the early stages of these processes and offer guidance for the development of effective prevention and intervention programs.

These results underscore the importance of the early identification of risk factors and their correlates to prevent adverse behavioral outcomes. A deeper understanding of these relationships can help parents, educators, and school professionals identify adolescents who may be at an increased risk for internalizing or externalizing behaviors. This knowledge supports efforts to cultivate positive and supportive relationships between parents, teachers, and students—an essential factor in predicting and mitigating risk behaviors.

Intervention programs that focus on enhancing self-esteem and strengthening adolescents' resistance to peer pressure may help reduce the harmful effects of psychological control and emotionally distant parenting. Such programs could be implemented in both family and school contexts, providing comprehensive support to at-risk youth. Additionally, the findings highlight the value of training initiatives for parents and educators to improve their skills in building nurturing and supportive environments that foster resilience and wellbeing.

By promoting awareness and collaborative intervention strategies, educational and parental systems can work together to create environments that not only identify at-risk individuals but also implement effective prevention measures. This proactive approach holds promise for reducing the incidence of risk behaviors and supporting healthier developmental trajectories.

Finally, these findings contribute to the broader field of developmental psychology by offering new insights into the mechanisms through which parenting influences adolescent adjustment, thereby guiding future research and practical applications.

## Limitations and future directions

It is important to note several limitations. First, this study is cross-sectional, and mediation analysis in such designs cannot establish causal relationships, as temporal ordering is essential for inferring causality. While the results are consistent with hypothesized pathways, the temporal ordering of variables cannot be confirmed, and causal interpretations should be made with caution. The associations observed between maternal psychological control and/or maternal warmth, self-esteem, peer susceptibility, and adolescent risk behaviors reflect correlations rather than causal effects. Without longitudinal data, it is not possible to determine the direction of these relationships or confirm whether changes in one variable lead to changes in another over time. Therefore, future research should examine these relationships and their potential causal pathways using longitudinal designs.

This study focused on how adolescents perceive their parents' behavior, specifically how critical or warm they believe their parents are perceived to be. These perceptions are shaped by the adolescents' emotional sensitivity or vulnerability. More emotionally sensitive adolescents may interpret the same parental behavior as more critical or less supportive than their less sensitive peers. Parents may not fully realize how their children interpret their behaviors or the potential emotional impact they may have on their children. Such discrepancies between parental intent and adolescent perception may be critical in understanding the development of emotional and behavioral outcomes during adolescence. Therefore, future research should examine and compare parents' and children's perceptions of parental behavior to determine the extent and nature of potential discrepancies between them. This also highlights the importance of considering individual differences in emotional reactivity when interpreting perceived parental behavior.

Another limiting factor of the study lies in the method of data collection itself. Students provided self-reports on how often they engaged in risk behaviors, which is the most common approach for gathering such data. However, these reports may be influenced by memory bias, underreporting due to the concealment of certain behaviors, or doubts regarding the anonymity of their responses. Future studies would benefit from incorporating multiple sources of information, such as teacher or parent reports, peer evaluations, or objective behavioral indicators, to increase validity and reduce potential response bias.

Additionally, it is essential to acknowledge that not all factors influencing the relationship between parenting and adolescent risk behaviors were examined. As is common in psychological research, human behavior and development are shaped by a wide range of influences, and a substantial portion of variance inevitably remains unexplained. In our study, the tested models accounted for approximately 20–35% of the variance, leaving considerable room for other potential determinants to influence the outcome. Future research should, therefore, incorporate additional factors that may play a meaningful role in this relationship. These include financial circumstances within the family, the quality of parental relationships, sibling dynamics, school climate, and relationships with teachers. For example, several studies have examined school climate and its connection to risk behaviors, showing that school culture and climate help shape students' and teachers' normative expectations (Deal and Peterson, 2016). Negative perceptions of school climate have been linked to higher involvement in undesirable behaviors, including substance use (DeWit et al., 2000), while favorable perceptions were associated with fewer suicidal and self-aggressive behaviors (Madjar et al., 2017). Other research also suggests that school-related factors, such as academic success and attitudes toward school, are closely tied to adolescent mental health (Denny et al., 2011; Patton et al., 2008).

Existing literature also emphasizes the relevance of individual characteristics (e.g., personality traits and locus of control), media exposure, the use of information and communication technologies, parental education, school attachment, and the quality of peer relationships. Taken together, these factors may contribute to a more comprehensive understanding of how parenting practices relate to the emergence of adolescent risk behaviors.

## Conclusion

The findings of this research suggest that self-esteem and susceptibility to peer pressure mediate the relationship between parental behavior and involvement in both externalizing and internalizing risk behaviors. However, these factors do not fully explain this relationship, suggesting that other influences are at play that were not examined in this study. Additionally, the research found that self-esteem acts as a mediator with a more significant role in the relationship between parental behavior and internalizing forms of risk behaviors compared to its role in externalizing risk behaviors. Peer pressure susceptibility emerged as a significant mediator for both types of risk behaviors.

## Data Availability

The raw data supporting the conclusions of this article will be made available by the authors, without undue reservation.

## References

[B1] Abbey A. Jacques A. Hayman L. W. Sobeck J. (2006). Predictors of early substance use among African American and Caucasian youth from urban and suburban communities. Merrill-Palmer Quart. 52, 305–326 doi: 10.1353/mpq.2006.0011

[B2] Achenbach T. M. (1966). The classification of children's psychiatric symptoms: a factor-analytic study. Psychol. Monogr.: Gen. Appl. 80, 1–37. doi: 10.1037/h0093906 5968338

[B3] Achenbach T. M. Dumenci L. Rescorla L. A. (2003). Are American children's problems still getting worse? A 23-year comparison. J. Abnor. Child Psychol. 31, 1–11. doi: 10.1023/A:1021700430364 12597695

[B4] Aguilar-Yamuza B. Herruzo-Pino C. Lucena-Jurado V. Raya-Trenas A. F. Pino-Osuna M. J. Chung M. (2023). Internalizing problems in childhood and adolescence: the role of the family. Alpha Psychiatry 24, 87–92. doi: 10.5152/alphapsychiatry.2023.221086 37440900 PMC10334679

[B5] Ajduković M. Ručević S. Šincek D. (2008). IstraŽivanje rasprostranjenosti rizičnog i delinkventnog ponašanja djece i mladih u urbanim sredinama - dodatni poticaj za ciljanu prevenciju. Dijete i društvo 10, 27–47. Available online at: https://sredisnjikatalogrh.gov.hr/srce-arhiva/28/72243/www.mobms.hr/media/19484/dijete%20i%20drustvo%20br%2010%201_2.pdf (Accessed February 26, 2025).

[B6] Aluko T. Daramola O. Omodehin A. (2024). The role of parenting styles and peer pressure in shaping risky behaviour tendencies among undergraduate students in Ogun State, Nigeria. NIU J. Soc. Sci. 10, 165–174. doi: 10.58709/niujss.v10i3.1991

[B7] Ãuranović M. Klasnić I. (2016). Povezanost školskog uspjeha i rizičnih ponašanja srednjoškolaca na internetu. Napredak 157, 263–281.

[B8] Baetens I. Claes L. Martin G. Onghena P. Grietens H. Van Leeuwen K. . (2014). Is non-suicidal self-injury associated with parenting and family factors? J. Early Adolesc. 34, 387–405. doi: 10.1177/0272431613494006

[B9] Barber B. K. (1996). Parental psychological control: revisiting a neglected construct. Child Dev. 67, 3296–3319. doi: 10.2307/1131780 9071782

[B10] Basili E. Zuffianò A. Pastorelli C. Thartori E. Lunetti C. Favini A. . (2021). Maternal and paternal psychological control and adolescents' negative adjustment: a dyadic longitudinal study in three countries. PLoS ONE 16:e0251437. doi: 10.1371/journal.pone.0251437 33989323 PMC8121295

[B11] Baumrind D. (1971). Current patterns of parental authority. Dev. Psychol. Monogr. 4, 1–103. doi: 10.1037/h0030372

[B12] Bean R. A. Bush K. R. McKenry P. C. Wilson S. M. (2003). The impact of parental support, behavioral control, and psychological control on the academic achievement and self-esteem of African American and European American adolescents. J. Adolesc. Res. 18, 523–541. doi: 10.1177/0743558403255070

[B13] Berc G. BlaŽeka Kokorić S. (2012). Family leisure as a factor of family cohesion and satisfaction with family life. Kriminologija Socijalna Integracija 20, 15–27. Available online at: https://hrcak.srce.hr/file/145666 (Accessed February 26, 2025).

[B14] Berk L. E. (2015). Dječja Razvojna Psihologija. Jastrebarsko: Naklada Slap.

[B15] Berndt T. J. (1979). Developmental changes in conformity to peers and parents. Dev. Psychol. 15, 608–616. doi: 10.1037/0012-1649.15.6.608

[B16] Biederman J. Faraone S. V. Hirshfeld-Becker D. R. Friedman D. Robin J. A. Rosenbaum J. F. (2001). Patterns of psychopathology and dysfunction in high-risk children of parents with panic disorder and major depression. Am. J. Psychiatry 158, 49–57. doi: 10.1176/appi.ajp.158.1.49 11136633

[B17] Bilsen J. (2018). Suicide and youth: risk factors. Front. Psychiatry 9:540. doi: 10.3389/fpsyt.2018.00540 30425663 PMC6218408

[B18] Blakemore S. J. Mills K. L. (2014). Is adolescence a sensitive period for socio-cultural processing? Annu. Rev. Psychol. 65, 187–207. doi: 10.1146/annurev-psych-010213-115202 24016274

[B19] Borra M. (2024). A study on peer pressure, loneliness, and self-esteem among school students. Int. J. Interdiscipl. Approach. Psychol. 2, 70–79. Available online at: https://www.psychopediajournals.com/index.php/ijiap/article/view/617 (Accessed February 26, 2025).

[B20] Bouillet D. Bijedić M. (2007). Rizična ponašanja učenika srednjih škola i doŽivljaj kvalitete razredno-nastavnog ozračja. Odgojne znanosti 9, 113–132. Available online at: https://hrcak.srce.hr/file/37102 (Accessed February 26, 2025).

[B21] Brown B. B. Clasen D. R. Eicher S. A. (1986). Perceptions of peer pressure, peer conformity dispositions, and self-reported behavior among adolescents. Dev. Psychol. 22, 521–530. doi: 10.1037/0012-1649.22.4.521

[B22] Brust Nemet M. Vrdoljak G. (2022). Gender (in)equality in child-rearing and housework between mothers and fathers. Croatian J. Educ. 24, 429–455. https://hrcak.srce.hr/clanak/406895 doi: 10.15516/cje.v24i2.4554

[B23] Campbell S. B. Shaw D. S. Gilliom M. (2000). Early externalizing behavior problems: toddlers and preschoolers at risk for later maladjustment. Dev. Psychopathol. 12, 467–488. doi: 10.1017/S0954579400003114 11014748

[B24] Champion L. A. Goodall G. Rutter M. (1995). Behaviour problems in childhood and stressors in early adult life. I. A 20 year follow-up of London school children. Psychol. Med. 25, 231–246. doi: 10.1017/S003329170003614X 7675912

[B25] Chan S. M. Chan K.-W. (2013). Adolescents' susceptibility to peer pressure: relations to parent-adolescent relationship and adolescents' emotional autonomy from parents. Youth Soc. 45, 286–302. doi: 10.1177/0044118X11417733

[B26] Chao R. K. (1994). Beyond parental control and authoritarian parenting style: understanding Chinese parenting through the cultural notion of training. Child Dev. 65, 1111–1119. doi: 10.2307/1131308 7956468

[B27] Chao R. K. (2001). Extending research on the consequences of parenting style for Chinese Americans and European Americans. Child Dev. 72, 1832–1843. doi: 10.1111/1467-8624.00381 11768148

[B28] Chen F. Garcia O. F. Alcaide M. Garcia-Ros R. Garcia F. (2024). Do we know enough about negative parenting? Recent evidence on parenting styles and child maladjustment. Psychol. Res. Behav. Manag. 16, 37–48. doi: 10.5093/ejpalc2024a4

[B29] Cohen J. (1988). Statistical Power Analysis for the Behavioral Sciences, 2. izdanje. Hillsdale, NJ: Lawrence Erlbaum.

[B30] Cruz S. Sousa M. Marchante M. Coelho V. A. (2023). Trajectories of social withdrawal and social anxiety and their relationship with self-esteem before, during, and after the school lockdowns. Sci. Rep. 13:16376. doi: 10.1038/s41598-023-43497-w 37773201 PMC10542336

[B31] Cvetković A. Todorović J. Janković I. (2023). “The relationship between risky and delinquent behavior, self-esteem and depression in adolescents,” in Psychological Applications and Trends 2023, eds. C. Pracana and M. Wang (Lisbon: Gima), 275–279. doi: 10.36315/2023inpact058

[B32] Deal T. E. Peterson K. D. (2016). Shaping School Culture. San Francisco, CA: Jossey Bass Publishers. doi: 10.1002/9781119210214

[B33] Deater-Deckard K. Dodge K. A. Bates J. E. Pettit G. S. (1996). Physical discipline among African American and European American mothers: links to children's externalizing behaviors. Dev. Psychol. 32, 1065–1072. doi: 10.1037/0012-1649.32.6.1065

[B34] Denny S. J. Robinson E. M. Utter J. Fleming T. M. Grant S. Milfont T. L. . (2011). Do schools influence student risk-taking behaviors and emotional health symptoms? J. Adolesc. Health 48, 259–267. doi: 10.1016/j.jadohealth.2010.06.020 21338897

[B35] DeWit D. J. Offord D. R. Sanford M. Rye B. J. Shain M. i Wright R. (2000). The effect of school culture on adolescent behavioural problems: self-esteem, attachment to learning, and peer approval of deviance as mediating mechanisms. Can. J. Sch. Psychol. 16, 15–38. doi: 10.1177/082957350001600102

[B36] Dobešová Cakirpaloglu S. Cech T. Štenclová V. (2020). ‘The relation of self-esteem on risk behaviour among emerging adolescents,” in EDULEARN20 Proceedings, eds. L. Gómez Chova, A. López Martínez, and I. Candel Torres (Palma Mallorca: International Academy of Technology, Education and Development), 3651–3657. doi: 10.21125/edulearn.2020.1010

[B37] Dopp A. R. Cain A. C. (2012). ‘The role of peer relationships in parental bereavement during childhood and adolescence. Death Stud. 36, 41–60. doi: 10.1080/07481187.2011.573175 24567994

[B38] Farrington D. P. (1997). “The relationship between low resting heart rate and violence,” in Biosocial Bases of Violence. Nato ASI Series, Vol. 292, eds. A. Raine, P. A. Brennan, D. P. Farrington, and S. A. Mednick (Boston, MA: Springer). doi: 10.1007/978-1-4757-4648-8_6

[B39] Farrington D. P. (1997). ?The relationship between low resting heart rate and violence,? in Biosocial Bases of Violence. Nato ASI Series, Vol. 292, eds. A. Raine, P. A. Brennan, D. P. Farrington, and S. A. Mednick (Boston, MA: Springer).

[B40] Fergusson D. M. Horwood L. J. Ridder E. M. Beautrais A. L. (2005). Subthreshold depression in adolescence and mental health outcomes in adulthood. Arch. Gen. Psychiatry 62, 66–72. doi: 10.1001/archpsyc.62.1.66 15630074

[B41] Field A. (2013). Discovering Statistics Using SPSS, 4th Edn. London: Sage.

[B42] Finkenauer C. Engels R. C. M. E. Baumeister R. F. (2005). Parenting behaviour and adolescent behavioural and emotional problems: the role of self-control. Int. J. Behav. Dev. 29, 58–69. doi: 10.1080/01650250444000333

[B43] Forko M. Lotar M. (2012). Izlaganje adolescenata riziku na nagovor vršnjaka – vaŽnost percepcije sebe i drugih. Kriminologija i socijalna integracija 20, 35–47. Available online at: https://hrcak.srce.hr/file/126644 (Accessed February 26, 2025).

[B44] Fortune S. Hetrick S. Hawton K. (2016). “Suicide and deliberate self-harm in children and adolescents,” in Rutter's Child and Adolescent Psychiatry, 6th Edn., eds. A. Thapar, D. S. Pine, J. F. Leckman, S. Scott, M. J. Snowling, and E. Taylor (Oxford: Wiley-Blackwell), 435–454.

[B45] Gould M. S. Fisher P. Parides M. Flory M. Shaffer D. (1996). Psychosocial risk factors of child and adolescent completed suicide. Arch. Gen. Psychiatry 53, 1155–1162. doi: 10.1001/archpsyc.1996.01830120095016 8956682

[B46] Guo J. Gao Q. Wu R. Ying J. You J. (2022). Parental psychological control, parent-related loneliness, depressive symptoms, and regulatory emotional self-efficacy: a moderated serial mediation model of non-suicidal self-injury. Arch. Suicide Res. 26, 1462–1477. doi: 10.1080/13811118.2021.1922109 34586982

[B47] Gvozdanovic A. Ilišin V. Adamovic M. Potocnik D. Baketa N. Kovacic M. (2019). Youth Study Croatia 2018/2019. Friedrich-Ebert-Stiftung. Available online at: https://library.fes.de/pdf-files/id-moe/15265.pdf (Accessed February 26, 2025).

[B48] Harter S. (1990). “Identity and self development,” in At the Threshold: The Developing Adolescent, eds. S. Feldman and G. Elliott (Cambridge, MA: Harvard University Press), 352–387.

[B49] Harter S. (1999). The Construction of the Self: A Developmental Perspective. New York, NY: Guilford.

[B50] Hayes A. F. (2013). Introduction to mediation, moderation, and conditional process analysis: A regression-based approach. New York, NY: The Guilford Press.

[B51] Hazan C. Shaver P. R. (1994). Attachment as an organizational framework for research on close relationships. Psychol. Inquiry 5, 1–22. doi: 10.1207/s15327965pli0501_1

[B52] Heilbron N. Prinstein M. J. (2008). ‘Peer influence and adolescent non-suicidal self-injury: a theoretical review of mechanisms and moderators. Appl. Prev. Psychol. 12, 169–177. doi: 10.1016/j.appsy.2008.05.004

[B53] Hesari N. K. Z. Hejazi E. (2011). The mediating role of self-esteem in the relationship between the authoritative parenting style and aggression. Proc. – Soc. Behav. Sci. 30, 1724–1730. doi: 10.1016/j.sbspro.2011.10.333

[B54] Hinnant J. B. Erath S. A. El-Sheikh M. (2015). Harsh parenting, parasympathetic activity and development of delinquency and substance use. J. Abnor. Psychol. 124, 137–151. doi: 10.1037/abn0000026 25688440 PMC4333737

[B55] Hinshaw S. P. (1987). On the distinction between attentional deficits/hyperactivity and conduct problems/aggression in child psychopathology. Psychol. Bull. 101, 443–463. doi: 10.1037/0033-2909.101.3.443 3602250

[B56] Hirsch B. DuBois D. (1991). ‘Self-esteem in early adolescence: the identification and prediction of contrasting longitudinal trajectories. J. Youth Adolesc. 20, 53–72. doi: 10.1007/BF01537351 24264916

[B57] Howell D. C. (2007). Statistical Methods for Psychology. Belmont, CA: Cengage Wadsworth.

[B58] Huang J. Zhang D. Chen Y. Yu C. Zhen S. Zhang W. (2022). Parental psychological control, psychological need satisfaction, and non-suicidal self-injury among Chinese adolescents: the moderating effect of sensation seeking. Child Youth Serv. Rev. 136:106417. doi: 10.1016/j.childyouth.2022.106417

[B59] Jackman D. M. MacPhee D. (2017). Self-esteem and future orientation predict adolescents' risk engagement. J. Early Adolesc. 37, 339–366. doi: 10.1177/0272431615602756

[B60] James S. A. (2013). Has Cutting Become Cool? (Ph.D. thesis). Massey University, Albany. Available online at: https://mro.massey.ac.nz/bitstreams/30996be3-6f6a-4903-a53b-a8451cf9694e/download (Accessed May 3, 2025).

[B61] Jessor R. (2016). The Origins and Development of Problem Behavior Theory. New York, NY: Springer. doi: 10.1007/978-3-319-40886-6

[B62] Jokić B. Ristić Dedić Z. (2023). Family Relations in the Republic of Croatia from the Perspective of Youth: Findings on the Occasion of the International Day of Families [Report]. Institute for Social Research. Available online at: https://wwwadmin.idi.hr/uploads/Obiteljski_odnosi_iz_perspektive_mladih_IDIZ_76843a8b83.pdf (Accessed May 2, 2025).

[B63] Kawabata Y. Alink L. R. Tseng W. van IJzendoorn M. H. Crick N. R. (2011). ‘Maternal and paternal parenting styles associated with relational aggression in children and adolescents: a conceptual analysis and meta-analytic review. Dev. Rev. 31, 240–278. doi: 10.1016/j.dr.2011.08.001

[B64] Kero A. (2022). Students' perceptions of father involvement in their upbringing: example of secondary school students in Zadar. Odgojno-obrazovne teme 5, 81–108. doi: 10.53577/oot.5.2.4

[B65] Khaleque A. (2017). Perceived parental hostility and aggression, and children's psychological maladjustment, and negative personality dispositions: a meta-analysis. J. Child Fam. Stud. 26, 977–988. doi: 10.1007/s10826-016-0637-9

[B66] Kim H. Parker J. G. Walker Marciano A. R. (2017). Interplay of self-esteem, emotion regulation, and parenting in young adolescents' friendship jealousy. J. Appl. Dev. Psychol. 52, 170–180. doi: 10.1016/j.appdev.2017.06.007

[B67] Klarin M. Ãerda V. (2014). Roditeljsko ponašanje i problemi u ponašanju kod adolescenata. Ljetopis socijalnog rada 21, 243–262. doi: 10.3935/ljsr.v21i2.17

[B68] Kliewer W. Fearnow M. D. Miller P. A. (1996). Family socialization of emotion expression and coping with peer provocation: implications for social competence. J. Child Psychol. Psychiatry 37, 945–954.

[B69] KneŽević Florić O. Pavlović A. Ninković S. (2021). Vršnjački pritisak i akademsko postignuće kao prediktori rizičnog ponašanja adolescenata'. Teme 44, 1123–1135. doi: 10.22190/TEME200712076K

[B70] Krauss S. Orth U. Robins R. W. (2020). ‘Family environment and self-esteem development: a longitudinal study from age 10 to 16. J. Pers. Soc. Psychol. 119, 457–478. doi: 10.1037/pspp0000263 31535888 PMC7080605

[B71] Leary M. R. (1999). Making sense of self-esteem. Curr. Direct. Psychol. Sci. 8, 32–35. doi: 10.1111/1467-8721.00008

[B72] Lebedina-Manzoni M. (2007). Psihološke osnove poremećaja u ponašanju. Jasrebarsko: Naklada Slap.

[B73] Lebedina-Manzoni M. Lotar M. Ricijaš N. (2008). Susceptibility to peer pressure among adolescents – challenges of definition and measurement. Ljetopis socijalnog rada 15, 401–419. Available online at: https://hrcak.srce.hr/file/49744 (Accessed February 26, 2025).

[B74] Lebedina-Manzoni M. Ricijaš N. (2013). Characteristics of youth regarding susceptibility to peer pressure. Kriminologija socijalna integracija 21, 39–48. Available online at: https://hrcak.srce.hr/109976 (Accessed February 26, 2025).

[B75] Liu J. (2004). Childhood externalizing behavior: theory and implication. J. Child Adolesc. Psychiatr. Nurs. 17, 93–103. doi: 10.1111/j.1744-6171.2004.tb00003.x 15535385 PMC1617081

[B76] Liu J. Chen X. Lewis G. (2011). Childhood internalizing behaviour: analysis and implications. J. Psychiatr. Mental Health Nurs. 18, 884–894. doi: 10.1111/j.1365-2850.2011.01743.x 22070805 PMC5675073

[B77] Livazović G. Ručević S. (2012). Rizični čimbenici eksternaliziranih ponašanja i odstupajućih navika hranjenja medu adolescentima. Društvena istraŽivanja: časopis za opća društvena pitanja 21, 733–752. doi: 10.5559/di.21.3.07

[B78] Loeber R. Farrington D. P. (2000). Young children who commit crime: epidemiology, developmental origins, risk factors, early interventions, and policy implications. Dev. Psychopathol. 12, 737–762. doi: 10.1017/S0954579400004107 11202042

[B79] Luk J. W. King K. M. McCarty C. A. McCauley E. Vander Stoep A. (2017). Prospective effects of parenting on substance use and problems across Asian/Pacific islander and European American youth: tests of moderated mediation. J. Stud. Alcohol Drugs 78, 521–530. doi: 10.15288/jsad.2017.78.521 28728634 PMC5551657

[B80] Maccoby E. E. Martin J. A. (1983). “Socialization in the context of the family: parent-child interaction,” in Handbook of Child Psychology: Socialization, Personality, and Social Development, eds. P. H. Mussen and E. M. Hetherington (New York, NY: Wiley), 1–101.

[B81] Macuka I. (2004). Skala percepcije obiteljskih odnosa. U K. Lackovic Grgin, A. Prorokovic, V. Cubela i Z. Penezic (Ur.), Zbirka psihologijskih skala i upitnika 2 (str. 17–21). Zadar: Filozofski fakultet.

[B82] Madjar N. Zalsman G. Ben Mordechai T. R. Shoval G. (2017). Repetitive vs. occasional non-suicidal self-injury and school-related factors among Israeli high school students. Psychiatry Res. 257, 358–360. doi: 10.1016/j.psychres.2017.07.073 28800516

[B83] Maglica T. DŽanko P. (2016). Internalised behavioural problems among high school students in Split. Školski vjesnik 65, 586. Available online at: https://hrcak.srce.hr/178257 (Accessed February 28, 2025).

[B84] Martínez-Casanova E. Molero-Jurado M. D. M. Pérez-Fuentes M. D. C. (2024). Self-esteem and risk behaviours in adolescents: a systematic review. Behav. Sci. 14:432. doi: 10.3390/bs14060432 38920764 PMC11201250

[B85] Miller S. Loeber R. Hipwell A. (2009). Peer deviance, parenting and disruptive behavior among young girls. J. Abnor. Child Psychol. 37, 139–152. doi: 10.1007/s10802-008-9265-1 18777132 PMC2680385

[B86] Modecki K. L. Uink B. (2018). Understanding Delinquency During the Teenage Years: Developmental Pathways of Antisocial Decision Making Among Disadvantaged Youth. Report to the Criminology Research Advisory Council Grant: CRG 13/14-15. Available online at: https://www.aic.gov.au/sites/default/files/2020-05/13-1415-FinalReport.pdf (Accessed May 5, 2025).

[B87] Mullan V. M. R. Golm D. Juhl J. Sajid S. Brandt V. (2023). The relationship between peer victimisation, self-esteem, and internalizing symptoms in adolescents: a systematic review and meta-analysis. PLoS ONE 18:e0282224. doi: 10.1371/journal.pone.0282224 36989220 PMC10058150

[B88] Nasiri Farsi T. Pali S. (2025). The impact of parental psychological control on adolescent risky behaviors with an emphasis on the mediating role of gratitude. J. Adolesc. Youth Psychol. Stud. 6, 70–80. doi: 10.61838/kman.jayps.6.1.8

[B89] Novak M. Maglica T. Radetić Paić M. (2022). School, family, and peer predictors of adolescent alcohol and marijuana use. Drugs: Educ. Prev. Policy 30, 486–496. doi: 10.1080/09687637.2022.2073869

[B90] Omisola E. T. Mosaku S. K. Fatunbi A. M. (2022). Influence of self-esteem and susceptibility to peer pressure on psychological wellbeing of in-school adolescents. Nigerian J. Behav. Stud. 1, 35–44. Available online at: https://njbs.fuoye.edu.ng/index.php/njbs/article/view/6 (Accessed February 26, 2025).

[B91] Orth U. Robins R. W. Roberts B. W. (2008). Low self-esteem prospectively predicts depression in adolescence and young adulthood. J. Pers. Soc. Psychol. 95, 695–708. doi: 10.1037/0022-3514.95.3.695 18729703

[B92] Osgood D. W. Ragan D. T. Wallace L. Gest S. D. Feinberg M. E. Moody J. (2013). Peers and the emergence of alcohol use: influence and selection processes in adolescent friendship networks. J. Res. Adolesc. 23, 500–512. doi: 10.1111/jora.12059 24307830 PMC3844135

[B93] Palacios I. Garcia O. F. Alcaide M. Garcia F. (2022). Positive parenting style and positive health beyond the authoritative: self, universalism values, and protection against emotional vulnerability from Spanish adolescents and adult children. Front. Psychol. 13:1066282. doi: 10.3389/fpsyg.2022.1066282 36591008 PMC9800864

[B94] Patton G. C. Olsson C. Bond L. Toumbourou J. W. Carlin J. B. Hemphill S. A. . (2008). Predicting female depression across puberty: Aatwo-nation longitudinal study. J. Am. Acad. Child Adolesc. Psychiatry 47, 1424–1432. doi: 10.1097/CHI.0b013e3181886ebe 18978636 PMC2981098

[B95] Peterson J. Freedenthal S. Sheldon C. Andersen R. (2008). Non-suicidal self-injury in adolescents. Psychiatry 5, 20–26. Available online at: https://pmc.ncbi.nlm.nih.gov/articles/PMC2695720/ (Accessed February 26, 2025). PMC2695720 19724714

[B96] Pettit G. S. Laird R. D. Dodge K. A. Bates J. E. Criss M. M. (2001). Antecedents and behavior-problem outcomes of parental monitoring and psychological control in early adolescence. Child Dev. 72, 583–598. doi: 10.1111/1467-8624.00298 11333086 PMC2766099

[B97] Pinquart M. Kauser R. (2018). Do the associations of parenting styles with behavior problems and academic achievement vary by culture? Results from a meta-analysis. Cult. Diver. Ethnic Min. Psychol. 24, 75–100. doi: 10.1037/cdp0000149 28394165

[B98] Portzky G. Audenaert K. van Heeringen K. (2005). Suicide among adolescents. A psychological autopsy study of psychiatric, psychosocial and personality-related risk factors. Soc. Psychiatry Psychiatr. Epidemiol. 40, 922–930. doi: 10.1007/s00127-005-0977-x 16217594

[B99] Prinstein M. J. Heilbron N. Guerry J. D. Franklin J. C. Rancourt D. Simon V. . (2010). ‘Peer influence and non-suicidal self-injury: longitudinal results in community and clinically-referred adolescent samples. J. Abnor. Child Psychol. 38, 669–682. doi: 10.1007/s10802-010-9423-0 20437255 PMC3686282

[B100] Raboteg-Šarić Z. Sakoman S. Brajša-Žganec A. (2002). ‘Parental child-rearing styles, leisure-time activities and youth risk behaviour. Društvena istraŽivanja 11, 239–263. Available online at: https://hrcak.srce.hr/19687 (Accessed February 26, 2025).

[B101] Raine A. Brennan P. Farrington D. Mednick S. A. (1997). Biosocial Basis of Violence. New York, NY: Plenum Press. doi: 10.1007/978-1-4757-4648-8

[B102] Reinherz H. Z. Giaconia R. M. Hauf A. M. C. Wasserman M. S. Silverman A. B. (1999). Major depression in the transition to adulthood: risks and impairments. J. Abnor. Psychol. 108, 500–510. doi: 10.1037/0021-843X.108.3.500 10466274

[B103] Rosenberg M. (1965). Society and the Adolescent Self-Image. Princeton, NJ: Princeton University Press. doi: 10.1515/9781400876136

[B104] Rosenberg M. Schooler C. Schoenbach C. (1989). Self-Esteem and adolescent problems: modeling reciprocal effects. Am. Sociol. Rev. 54, 1004–1018. doi: 10.2307/2095720

[B105] Rothenberg W. A. Lansford J. E. Bornstein M. H. Chang L. Deater-Deckard K. Di Giunta L. . (2020). Effects of parental warmth and behavioral control on adolescent externalizing and internalizing trajectories across cultures. J. Res. Adolesc. 30, 835–855. doi: 10.1111/jora.12566 32609411 PMC8059478

[B106] Ručević S. (2011). Povezanost privrŽenosti roditeljima s rizičnim i delinkventnim ponašanjem kod adolescenata. Društvena istraŽivanja: časopis za opća društvena pitanja 20, 167–187. doi: 10.5559/di.20.1.09

[B107] Ručević S. Ajduković M. Šincek D. (2009). Development of youth self-reported delinquency and risk behaviors questionnaire (SRDP-2007). Kriminol. Socijalna Integr. 17, 1–11. Available online at: https://hrcak.srce.hr/40729 (Accessed February 26, 2025).

[B108] Shek D. T. L. (2007). A longitudinal study of perceived parental psychological control and psychological well-being in Chinese adolescents in Hong Kong. J. Clin. Psychol. 63, 1–22. doi: 10.1002/jclp.20331 17115428

[B109] Silk J. S. Morris A. S. Kanaya T. Steinberg L. (2003). Psychological control and autonomy granting: opposite ends of a continuum or distinct constructs? J. Res. Adolesc. 13, 113–128. doi: 10.1111/1532-7795.1301004

[B110] Šimić Šašić S. Klarin M. (2009). Varanje u srednjim školama u Hrvatskoj i u Bosni i Hercegovini. Društvena istraŽivanja 18, 999–1022.Available online at: https://hrcak.srce.hr/45779 (Accessed February 26, 2025).

[B111] Smetana J. G. Daddis C. (2002). Domain-specific antecedents of parental psychological control and monitoring: the role of parenting beliefs and practices. Child Dev. 73, 563–580. doi: 10.1111/1467-8624.00424 11949909

[B112] Symeou M. Georgiou S. (2017). Externalizing and internalizing behaviours in adolescence, and the importance of parental behavioural and psychological control practices. J. Adolesc. 60, 104–113 doi: 10.1016/j.adolescence.2017.07.007 28841442

[B113] Szcześniak M. Bielecka G. Madej D. Pieńkowska E. Rodzeń W. (2020). The role of self-esteem in the relationship between loneliness and life satisfaction in late adulthood: evidence from Poland. Psychol. Res. Behav. Manag. 13, 1201–1212. doi: 10.2147/PRBM.S275902 33363419 PMC7754268

[B114] Tabachnick B. G. Fidell L. S. (2013). Using Multivariate Statistics, 6 Edn. Boston, MA: Pearson.

[B115] Tian L. Dong X. Xia D. Liu L. Wang D. (2020). Effect of peer presence on adolescents' risk-taking is moderated by individual self-esteem: an experimental study. Int. J. Psychol. 55, 373–379. doi: 10.1002/ijop.12611 31339180

[B116] Tørmoen A. J. Myhre M. Walby F. A. Grøholt B. Rossow I. (2020). Change in prevalence of self-harm from 2002 to 2018 among Norwegian adolescents. Eur. J. Public Health 30, 1–5. doi: 10.1093/eurpub/ckaa042 32134469 PMC7445045

[B117] Toth S. L. Cicchetti D. (1996). Patterns of relatedness, depressive symptomatology, and perceived competence in maltreated children. J. Consult. Clin. Psychol. 64, 32–41. doi: 10.1037/0022-006X.64.1.32 8907082

[B118] Tschan T. Schmid M. In-Albon T. (2015). Parenting behavior in families of female adolescents with nonsuicidal self-injury in comparison to a clinical and a nonclinical control group. Child Adolesc. Psychiatry Ment. Health 9:17. doi: 10.1186/s13034-015-0051-x 26157478 PMC4495632

[B119] Tully E. C. Iacono W. G. McGue M. (2008). An adoption study of parental depression as an environmental liability for adolescent depression and childhood disruptive disorders. Am. J. Psychiatry 165, 1148–1154. doi: 10.1176/appi.ajp.2008.07091438 18558644 PMC2573034

[B120] Vieno A. Nation M. Pastore M. Santinello M. (2009). Parenting and antisocial behavior: a model of the relationship between adolescent self-disclosure, parental closeness, parental control, and adolescent antisocial behavior. Dev. Psychol. 45, 1509–1519. doi: 10.1037/a0016929 19899910

[B121] White H. V. Silamongkol T. Wiglesworth A. Labella M. H. Goetz E. R. Cullen K. R. . (2021). Maternal emotion socialization of adolescent girls engaging in non-suicidal self-injury. Res. Child Adolesc. Psychopathol. 49, 683–695. doi: 10.1007/s10802-020-00758-w 33521893 PMC8443321

[B122] Wu Y. Yuan R. Wu Y. (2022). Good can be stronger than bad: the daily relationship among maternal warmth, mother-teen conflict and adolescents' self-esteem. Curr. Psychol. 42, 25745–25754. doi: 10.1007/s12144-022-03718-3 36068882 PMC9436732

[B123] Yates T. M. (2004). The developmental psychopathology of self-injurious behavior: compensatory regulation in posttraumatic adaptation. Clin. Psychol. Rev. 24, 35–74. doi: 10.1016/j.cpr.2003.10.001 14992806

[B124] Zelenika T. Vekić T. Tadić-Lesko K. (2024). Association of sociodemographic characteristics with externalized behavioral disorders in adolescents. Acta Iadertina 21, 31–58. doi: 10.15291/ai.4511

[B125] Zhang L. Wang R. Chen L. (2024a). The impact of harsh parental discipline and emotional warmth on adolescent problem behaviors. Psychol. Res. Behav. Manag. 17, 2309–2319. doi: 10.2147/PRBM.S466830 38860193 PMC11164210

[B126] Zhang L. Wang R. Li Y. Chen L. (2024b). The impact of maternal emotional warmth on adolescents' internalizing problem behaviors: the roles of meaning in life and friendship conflict. Front. Psychol. 15:1478610. doi: 10.3389/fpsyg.2024.1478610 39679149 PMC11638585

[B127] Zimmerman M. Copeland L. Shope J. T. Dielman T. E. (1997). A longitudinal study of self-esteem: implications for adolescent development. J. Youth Adolesc. 26, 117–141. doi: 10.1023/A:1024596313925

[B128] ŽiŽak A. Koller-Trbović N. Jedud I. (2004). “Behavioral disorders in children and youth: the perspective of experts and the perspective of children and youth,” in Behavioral Disorders and Risky Behaviors: Approaches and Conceptual Definitions, eds. J. Bašić, N. Koller-Trbović, and S. Uzelac (Zagreb: Faculty of Education and Rehabilitation Sciences, University of Zagreb) 119–139.

