# Supplementary material for: Self-esteem and peer pressure susceptibility mediating the link between maternal behavior and adolescent risk behaviors
Source: Front Psychol. 2025 Nov 6;16:1655371. doi: 10.3389/fpsyg.2025.1655371 (PMC12631241; doi:10.3389/fpsyg.2025.1655371)
Supplement: Supplementary file 1 [file Data_Sheet_1.pdf]

Supplementary Material

Test of the mediating effects of self-esteem and peer pressure susceptibility between paternal psychological control and mild externalizing risk behaviors

TABLE S1 Regression model of the effect of paternal psychological control on adolescents' mild externalizing risk behaviors.

Variables	*β*	*t*	*p*	R^2	*F*
Step 1 outcome variable: Self-esteem					
Control variable Gender	-.25	-5.48	.00	.18	42.24*
Predictor Psychological control	-.30	-6.52	.00		
Step 2 outcome variable: Peer pressure susceptibility					
Control variable Gender	.04	.72	.47	.15	23.78*
Predictor Psychological control	.28	5.56	.00		
Mediator 1 Self-esteem	-.19	-3.67	.00		
Step 3 outcome variable: mild externalizing risk behaviors					
Control variable Gender	-.05	-1.15	.25	.22	27.76*
Predictor Psychological control	.20	4.01	.00		
Mediator 1 Self-esteem	-.01	-.24	.81		
Mediator 2 Peer pressure susceptibility	.37	7.53	.00		

*p<.001

TABLE S2 Multiple mediating effect analysis of paternal psychological control on adolescents' mild externalizing risk behaviors.

	Effect	SE	Bootstrap 95% CI		Effect ratio (%)
			Low	High	
Total effect	.035	.005	.024	.045	100%
Direct effect	.021	.005	.010	.032	60%
	Effect	Boot SE	Bootstrap 95% CI		Effect ratio (%)
			BootLow	BootHigh	
Total indirect effect	.013	.003	.008	.019	37.1%
Path 1: Maternal psychological control → Self-esteem → Mild externalizing risk behaviors	.0004	.002	-.003	.004	1.1%
Path 2: Maternal psychological control → Peer pressure susceptibility → Mild externalizing risk behaviors	.011	.003	.006	.016	31.4%
Path 3: Maternal psychological control → Self-esteem → Peer pressure susceptibility → Mild externalizing risk behaviors	.002	.001	.001	.004	5.7%

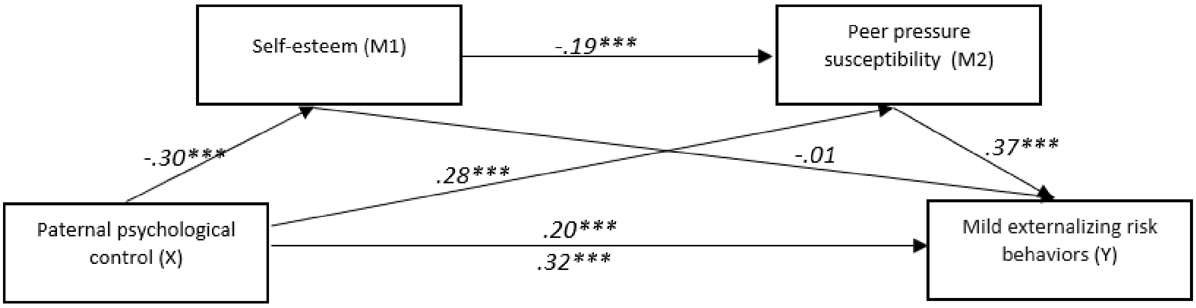

FIGURE S1 The mediating effect model of self-esteem and peer pressure susceptibility on the relationship between paternal psychological control and mild externalizing risk behaviors.

The total indirect effect of paternal psychological control on mild externalizing risk behaviors was significant, abcs = .13, 95% CI [.07, .18]. Examination of the specific indirect effects showed that the pathway through peer pressure susceptibility was the strongest and significant (abcs = .10, 95% CI [.06, .15]), followed by the sequential pathway through self-esteem and peer pressure susceptibility (abcs = .02, 95% CI [.01, .04]). The indirect effect via self-esteem alone was not significant (abcs = .004, 95% CI [−.03, .04]). These findings indicate that the effect of peer pressure susceptibility on mild externalizing risk behaviors is transmitted through peer pressure susceptibility, both directly and in sequence with self-esteem.

TABLE S3 Regression model of the effect of paternal emotional warmth on adolescents' mild externalizing risk behaviors.

Variables	*β*	*t*	*p*	R^2	*F*
Step 1 outcome variable: Self-esteem					
Control variable Gender	-.21	-4.69	.00	.27	74.09*
Predictor Emotional warmth	.44	10.03	.00		
Step 2 outcome variable: Peer pressure susceptibility					
Control variable Gender	.03	0.62	.54	.14	20.37*
Predictor Emotional warmth	-.25	-4.65	.00		
Mediator 1 Self-esteem	-.16	-2.93	.004		
Step 3 outcome variable: Mild externalizing risk behaviors					
Control variable Gender	-.06	-1.21	.23	.21	25.91*
Predictor Emotional warmth	-.17	-3.18	.002		
Mediator 1 Self-esteem	.01	0.13	.90		
Mediator 2 Peer pressure susceptibility	.38	7.92	.00		

*p<.001

TABLE S4 Multiple mediating effect analysis of paternal psychological control on adolescents' mild externalizing risk behaviors.

	Effect	SE	Bootstrap 95% CI		Effect ratio (%)
			Low	High	
Total effect	-.018	.003	-.024	-.012	100%
Direct effect	-.011	.003	-.017	-.004	61.1%
	Effect	Boot SE	Bootstrap 95% CI		Effect ratio (%)
			BootLow	BootHigh	
Total indirect effect	-.008	.002	-.012	-.004	44.4%
Path 1: Maternal emotional warmth → Self-esteem → Mild externalizing risk behaviors	.0002	.002	-.003	.004	0%
Path 2: Maternal emotional warmth → Peer pressure susceptibility→Mild externalizing risk behaviors	-.006	.002	-.009	-.003	33.3%
Path 3: Maternal emotional warmth → Self-esteem → Peer pressure susceptibility → Mild externalizing risk behaviors	-.002	.001	-.003	-.001	11.1%

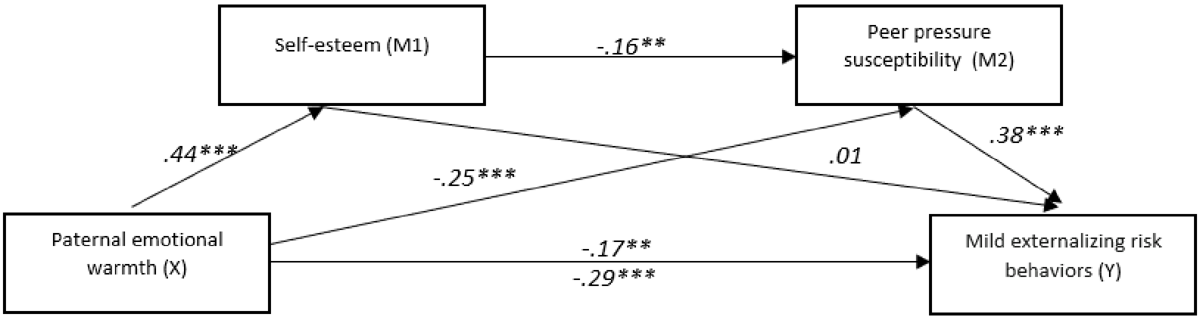

FIGURE S2 The mediating effect model of self-esteem and peer pressure susceptibility on the relationship between paternal emotional warmth and mild externalizing risk behaviors.

The analysis revealed a significant total indirect effect of paternal emotional warmth on mild externalizing risk behaviors, abcs = −.12, 95% CI [−.19, −.06]. Among the specific pathways, the indirect effect through peer pressure susceptibility was the strongest and significant, abcs = −.10, 95% CI [−.15, −.05]. A more minor but still significant sequential effect was observed through self-esteem and peer pressure susceptibility, abcs = −.03, 95% CI [−.05, −.01]. In contrast, the indirect effect through self-esteem alone was not significant, abcs = .003, 95% CI [−.04, .05]. These findings suggest that the impact of paternal emotional warmth on mild externalizing risk behaviors is primarily transmitted through peer pressure susceptibility, with self-esteem contributing only when operating in sequence with peer pressure susceptibility.

Test of the mediating effects of self-esteem and peer pressure susceptibility between paternal psychological control and internalizing risk behaviors

TABLE S5 Regression model of the effect of paternal psychological control on adolescents' internalizing risk behaviors.

Variables	β	t	p	R^2	F
Step 1 outcome variable: Self-esteem					
Control variable Gender	-.26	-5.56	.00	.18	42.54*
Predictor Psychological control	-.30	-6.51	.00		
Step 2 outcome variable: Peer pressure susceptibility					
Control variable Gender	.03	.63	.53	.15	22.99*
Predictor Psychological control	.27	5.47	.00		
Mediator 1 Self-esteem	-.19	-3.65	.00		
Step 3 outcome variable: Internalizing risk behaviors					
Control variable Gender	.12	2.68	.008	.33	47.07*
Predictor Psychological control	.22	4.86	.00		
Mediator 1 Self-esteem	-.29	-6.16	.00		
Mediator 2 Peer pressure susceptibility	.20	4.42	.00		

*p<.001

TABLE S6 Multiple mediating effect analysis of paternal psychological control on adolescents' internalizing risk behaviors.

	Effect	SE	Bootstrap 95% CI		Effect ratio (%)
			Low	High	
Total effect	.050	.006	.038	.061	100%
Direct effect	.03	.006	.018	.041	60.0%
	Effect	Boot SE	Bootstrap 95% CI		Effect ratio (%)
			BootLow	BootHigh	
Total indirect effect	.020	.004	.013	.028	40.0%
Path 1: Paternal psychological control → Self-esteem → Internalizing risk behaviors	.011	.003	.006	.018	22.0%
Path 2: Paternal psychological control → Peer pressure susceptibility → Internalizing risk behaviors	.007	.003	.002	.013	14.0%
Path 3: Paternal psychological control →Self-esteem → Peer pressure susceptibility → Internalizing risk behaviors	.002	.001	.0003	.003	4.0%

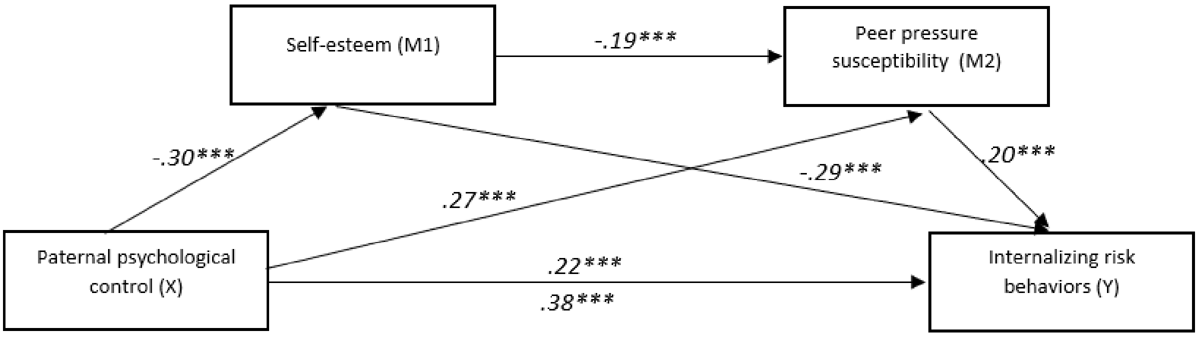

FIGURE S3 The mediating effect model of self-esteem and peer pressure susceptibility on the relationship between paternal psychological control and internalizing risk behaviors.

Analyses of indirect effects further support these findings. The total indirect effect of psychological control on internalizing risk behaviors was significant (abcs = .15, 95% CI [.10, .21]), reflecting a medium effect size (Cohen, 1988). The strongest indirect pathway was via self-esteem (abcs = .09, 95% CI [.05, .13]), followed by the path via peer pressure susceptibility (abcs = .05, 95% CI [.02, .09]). A more minor but significant sequential indirect effect was also observed through both self-esteem and peer pressure susceptibility (abcs = .01, 95% CI [.003, .02]).

Test of the mediating effects of self-esteem and peer pressure susceptibility between paternal emotional warmth and internalizing risk behaviors

TABLE S7 Regression model of the effect of paternal emotional warmth on adolescents' internalizing risk behaviors.

Variables	*β*	*t*	*p*	R^2	*F*
Step 1 outcome variable: self-esteem					
Control variable Gender	-.21	-4.75	.00	.28	75.09*
Predictor: emotional warmth	.44	10.09	.00		
Step 2 outcome variable: Peer pressure susceptibility					
Control variable Gender	.03	.53	.60	.13	19.60*
Predictor: emotional warmth	-.25	-4.54	.00		
Mediator 1: self-esteem	-.16	-2.91	.004		
Step 3 outcome variable: Internalizing risk behaviors					
Control variable Gender	.11	2.50	.01	.34	50.53*
Predictor emotional warmth	-.28	-5.79	.00		
Mediator 1 self - esteem	-.23	-4.69	.00		
Mediator 2 Peer pressure susceptibility	.20	4.55	.00		

*p<.001

TABLE S8 Multiple mediating effect analysis of paternal emotional warmth on adolescents' internalizing risk behaviors.

	Effect	SE	Bootstrap 95% CI		Effect ratio (%)
			Low	High	
Total effect	-.035	.004	-.042	-.028	100%
Direct effect	-.022	.004	-.029	-.015	62.9%
	Effect	Boot SE	Bootstrap 95% CI		Effect ratio (%)
			BootLow	BootHigh	
Total indirect effect	-.013	.003	-.018	-.008	37.1%
Path 1: Paternal emotional warmth → Self-esteem → Internalizing risk behaviors	-.008	.002	-.012	-.004	2.9%
Path 2: Paternal emotional warmth → Peer pressure susceptibility → Internalizing risk behaviors	-.004	.002	-.007	-.001	11.4%
Path 3: Paternal emotional warmth → Self-esteem → Peer pressure susceptibility → Internalizing risk behaviors	-.001	.001	-.002	-.0002	2.9%

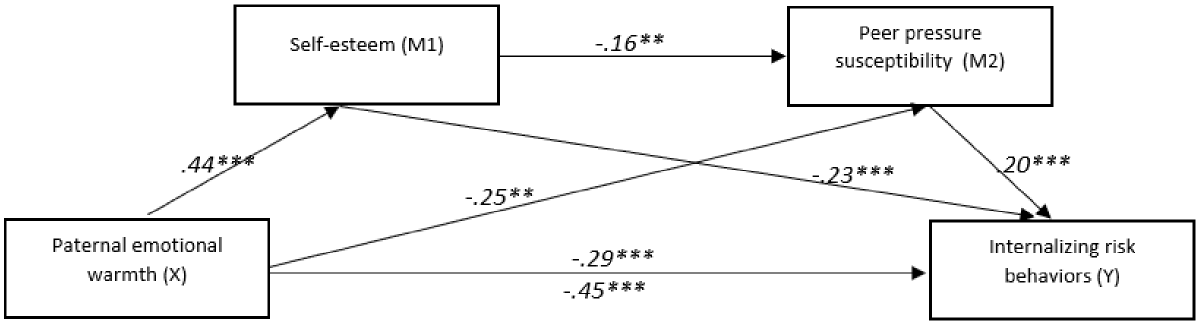

FIGURE S4 The mediating effect model of self-esteem and peer pressure susceptibility on the relationship between paternal emotional warmth and internalizing risk behaviors.

Analyses of indirect effects confirmed that the total indirect effect of emotional warmth on internalizing risk behaviors was significant (abcs = –.17, 95% CI [–.22, –.10]). The strongest pathway was through self-esteem (abcs = –.10, 95% CI [–.16, –.05]), followed by the path through peer pressure susceptibility (abcs = –.05, 95% CI [–.09, –.02]). A more minor but significant sequential indirect effect was also detected, operating first through self-esteem and then through peer pressure susceptibility (abcs = –.01, 95% CI [–.03, –.003]). The contrasts between indirect effects further indicated that the pathway via self-esteem was significantly stronger than both the pathway via peer pressure susceptibility and the sequential path. In addition, the pathway via peer pressure susceptibility alone was stronger than the sequential pathway.
